# Host contributes to longitudinal diversity of fecal microbiota in swine selected for lean growth

**DOI:** 10.1186/s40168-017-0384-1

**Published:** 2018-01-04

**Authors:** Duc Lu, Francesco Tiezzi, Constantino Schillebeeckx, Nathan P. McNulty, Clint Schwab, Caleb Shull, Christian Maltecca

**Affiliations:** 1 Department of Animal Science, North Carolina State University, Raleigh, 27606 NC USA; 2 Matatu Inc., 4320 Forest Park Ave., Suite 321, Saint Louis, 63108 MO USA; 3 The Maschhoffs LLC., Carlyle, 62231 IL USA

**Keywords:** Fecal microbiome, Longitudinal changes, Diversity, Genetic parameters

## Abstract

**Background:**

In pigs, gut bacteria have been shown to play important roles in nutritional, physiological, and immunological processes in the host. However, the contribution of their metagenomes or part of them, which are normally reflected by fragments of 16S rRNA-encoding genes, has yet to be fully investigated.

**Results:**

Fecal samples, collected from a population of crossbred pigs at three time points, including weaning, week 15 post weaning (hereafter “week 15”), and end-of-feeding test (hereafter “off-test”), were used to evaluate changes in the composition of the fecal microbiome of each animal over time. This study used 1205, 1295, and 1283 samples collected at weaning, week 15, and off-test, respectively. There were 1039 animals that had samples collected at all three time points and also had phenotypic records on back fat thickness (BF) and average daily body weight gain (ADG). Firmicutes and Bacteroidetes were the most abundant phyla at all three time points. The most abundant genera at all three time points included *Clostridium*, *Escherichia*, *Bacteroides*, *Prevotella*, *Ruminococcus*, *Fusobacterium*, *Campylobacter*, *Eubacterium*, and *Lactobacillus*. Two enterotypes were identified at each time point. However, only enterotypes at week 15 and off-test were significantly associated with BF*.* We report herein two novel findings: (i) alpha diversity and operational taxonomic unit (OTU) richness were moderately heritable at week 15, *h*^2^ of 0.15 ± 0.06 to 0.16 ± 0.07 and 0.23 ± 0.09 to 0.26 ± 0.08, respectively, as well as at off-test, *h*^2^ of 0.20 ± 0.09 to 0.33 ± 0.10 and 0.17 ± 0.08 to 0.24 ± 0.08, respectively, whereas very low heritability estimates for both measures were detected at weaning; and (ii) alpha diversity at week 15 had strong and negative genetic correlations with BF, − 0.53 ± 0.23 to − 0.45 ± 0.25, as well as with ADG, − 0.53 ± 0.32 to − 0.53 ± 0.29.

**Conclusions:**

These results are important for efforts to genetically improve the domesticated pig because they suggest fecal microbiota diversity can be used as an indicator trait to improve traits that are expensive to measure.

**Electronic supplementary material:**

The online version of this article (10.1186/s40168-017-0384-1) contains supplementary material, which is available to authorized users.

## Background

Until recently, research in physiology and production in livestock has focused on understanding individual’s variability for a wide array of traits. With the assistance of DNA technology, research in swine genetics has changed to include the use of single nucleotide polymorphisms (SNP) in identifying causative mutations that underpin variation in phenotypic measures, as well as to predict future performance of the pig, using all the genes or subsets of genes across the animal genome. However, the pig genome contains less than half the number of genes existing in its second genome, which is its microbiome [1] whose impact on the host has yet to be fully investigated.

The literature documents a growing body of research characterizing the microbiomes of pigs at different stages of development, including early life [2–5], growing stage [6, 7], and later stage [8, 9]. Bacteria in the pig gut have been shown to impact host nutritional, physiological, and immunological processes in various ways [10–15]. Though gut microbial diversity in pigs has been described to some extent [8, 9], most of the previous studies have been characterized by small sample sizes and/or by targeted manipulation of specific groups of bacteria in the gut at specific times in the animals’ lives, often in relation to nutrition studies. Composition and function of a healthy microbial ecosystem however have yet to be qualitatively and quantitatively defined to be used as a tool to maximize animal health and performance. Particularly, microbiome diversity has not been studied at large scales, including large sample sizes being conducted through several stages of the production life of the pig. Additionally, the impact of diversity has not been investigated from a perspective that could be used to proactively predict and manipulate health and/or performance of the host.

Studies in domestic pigs have revealed how outbred pigs carry an amount of genetic variation comparable to that of outbred human populations [16] and are more similar to humans than rodents in terms of anatomy, genetics, and physiology [17]. Pigs also have similar clinical manifestations and susceptibility to many enteric pathogens detected in humans [18–22]. With regard to the gut microbes, 96% of the functional pathways found in the human catalog are also present in the pig catalog [1]. Therefore, the pig model of human health studies, especially in gut microbiome, has drawn the attention of the research community.

The research reported herein is part of a larger project aimed at investigating the use of microbial information to improve pig health and production, including higher efficiency of feed utilization, better meat quality, and healthier pigs. Within this paper, we focused on two main objectives: (1) characterizing temporal changes in the microbiome community of pig feces with respect to both composition and diversity; and (2) investigating the potential influence of host genetics on this diversity.

## Methods

### Animals

The pigs used in this study were raised in a commercial setting operated by The Maschhoffs, LLC (Carlyle, IL, USA); therefore, animal use approval was not needed for the collection of these data. Twenty-eight purebred Duroc sires, from a Duroc population under selection for lean growth, were crossed with Large White × Landrace or Landrace × Large White sows (dam lines) to produce the offspring that were used in this study. The pigs were weaned at 18.6 ± 1.09 days old and were moved to a nursery-finishing facility, where they were put in groups of 20 individuals per pen. Pen mates were paternal half-siblings of the same gender and of similar weaning weight. The experiment was repeated 6 times, each of which comprised of 2 pens (1 pen of female pigs and 1 pen of castrated male pigs that are referred to “male” hereafter) from each of the 28 sires. Pigs that came together in 1 replicate were put in 1 contemporary group (hereafter “cg”) in analyses that followed.

The test period began the day the pigs were moved to the nursery-finishing facility. During the nursery, growth, and finish periods, they were fed standard pelleted feed. During the grow-finish period, they were fed standard diets, which were based on sex and live weight. Details of diet formulae and their nutritional values are provided (see Additional file 1). The pigs received a standard vaccination and medication routine (see Additional file 2). End of test (hereafter “off-test”) was reached on a pen-specific basis when all pigs in a pen achieved an average live weight of 136 kg and were harvested. Their average age at harvest was 196.4 ± 7.86 days.

Rectal swabs were collected from all pigs in a pen at 3 time points, including weaning, 15 weeks post weaning (average 118.2 ± 1.18 days, hereafter “week 15”), and off-test. Four pigs were chosen randomly per pen for lean carcass growth measurements, and their rectal swabs were used for microbiome sequencing. In the end, the number of samples at weaning, week 15, and off-test were 1205, 1295, and 1283, respectively. There were 1039 animals having samples collected at all 3 time points. More details on the distribution of samples across families, time points, and sex are provided (see Additional file 3).

Back fat thickness was recorded on live animals at weeks 18 and 22 post weaning, hereafter referred to as BF_18 and BF_22, respectively. Live weights were recorded at weaning as well as weeks 14 and 22 post weaning and were used to compute average daily body weight gain from weaning to week 14 (hereafter “ADGw_14”) and from week 14 through week 22 (hereafter “ADG14_22”).

### DNA extraction and purification

Total DNA (gDNA) was extracted from each rectal swab by mechanical disruption in phenol:chloroform. Briefly, 650 μL of extraction buffer (200 mM Tris; 200 mM NaCl; 20 mM EDTA, pH 8.0) was added to each swab stored in a 2-mL self-standing screw cap tube (Axygen, CA, USA). Tubes were shaken using a Mini-BeadBeater-96 (MBB-96; BioSpec, OK, USA) for 20 s to free sample material from the swab head. Following a brief centrifugation (10 s; 500 × *g*) to pull down any dislodged material, each swab head was removed from its tube using sterile forceps. Samples were frozen solid at − 80 °C, and approximately 250 μL of 0.1 mm zirconia/silica beads (BioSpec) and a 3.97 mm stainless steel ball were added to the sample (while still frozen to avoid splashing). Samples were allowed to thaw briefly, after which 210 μL 20% SDS and 500 μL phenol:chloroform:IAA (25:24:1, pH 8.0) were added. Bead-beating was performed on the MBB-96 (4 min, room temperature), samples were centrifuged (3220 × *g*; 4 min), and 250 μL of the aqueous phase was transferred to a new tube. One hundred microliters of this crude DNA was then further purified using a QIAquick 96 PCR purification kit (Qiagen, MD, USA). Purification was performed per the manufacturer’s instructions with the following minor modifications: (i) sodium acetate (3 M, pH 5.5) was added to Buffer PM to a final concentration of 185 mM to ensure optimal binding of genomic DNA to the silica membrane; (ii) crude DNA was combined with 4 volumes of Buffer PM (rather than 3 volumes); and, (iii) DNA was eluted in 100 μL Buffer EB (rather than 80 μL).

### Illumina library preparation and sequencing

Phased, bi-directional amplification of the V4 region (515–806) of the 16S rRNA gene was employed to generate indexed libraries for Illumina sequencing using the strategy described in [23]. Amplicon libraries were quantified using the Qubit dsDNA assay kit (Thermo Fisher Scientific Inc., MA, USA) before being pooled in equimolar ratios. These final pools were purified using Agencourt AMPure XP beads (Beckman Coulter) per the manufacturer’s instructions. Purified pools were supplemented with 5–10% PhiX control DNA and were sequenced on an Illumina MiSeq machine as paired-end 2 × 250 + 13 bp index reactions using the 600v3 kit. Un-demultiplexed FASTQ files were generated by MiSeq Reporter. All sequencing was performed at the DNA Sequencing Innovation Lab at the Center for Genome Sciences and Systems Biology at Washington University in St. Louis.

### 16S rRNA gene sequencing and quality control of data

Pairs of V4 16S rRNA gene sequences were first merged into a single sequence using FLASh v1.2.11 [24], with a required overlap of at least 100 and not more than 250 base pairs in order to provide a confident overlap. Sequences with a mean quality score below Q35 were then filtered out using PRINSEQ v0.20.4 [25]. Sequences were oriented in the forward direction and any primer sequences were matched and trimmed off; during primer matching, up to 1 mismatch was allowed. Sequences were subsequently demultiplexed using QIIME v1.9 [26]. Sequences with > 97% nucleotide sequence identity were then clustered into operational taxonomic units (hereafter “OTUs”) using QIIME with the following settings: max_accepts = 50, max_rejects = 8, percent_subsample = 0.1 and --suppress_step4. A modified version of Greengenes [27–29] was used as the reference database. Input sequences that had 10% of the reads with no hit to the reference database were then clustered de novo with UCLUST [30] to generate new reference OTUs to which the remaining 90% of reads were assigned. The most abundant sequence in each cluster was used as the representative sequence for the OTU. Sparse OTUs were then filtered out by requiring a minimum total observation count of 1200 for an OTU to be retained, and the OTU table was rarefied to 10,000 counts per sample. Average good’s coverage estimates for samples at weaning, week 15 and off-test were 0.99 ± 0.002, 0.98 ± 0.002, and 0.98 ± 0.002, respectively. Finally, the Ribosomal Database Project (RDP) classifier (v2.4) was retrained in the manner described in [31] with 0.8 cutoff used to assign taxonomy to the representative sequences.

### Comparative analysis of microbiome composition

In order to compare microbiome composition longitudinally, relative abundance counts were logarithm-transformed and zero-centered, then plugged into Kruskal-Wallis test. Adjustment of *P* values for multiple testing was completed via Bonferroni correction. This comparative analysis was performed at the genus and species levels.

### Clustering analysis

Clustering analysis in this paper was performed in two parts. The first part was aimed to investigate whether or not samples at the three time points could separate from one another based on their microbiome compositions. OTU counts were divided by the total count for each sample (which was 10,000), logarithm-transformed, and zero-centered before being applied to the R function “prcomp” for principal component analysis. This analysis was performed at the phylum, class, order, family, genus, and species levels. The results are presented in Fig. 2.

Part 2 of the clustering analysis was focused on identifying enterotypes among the samples collected at each time point. For that purpose, Jensen-Shannon Divergence (hereafter “JSD”) [32] was calculated at three separate time points according to the relative abundance of each genus in each sample using the “dist.JSD” function coded in R [33]. Based on the obtained distance matrix, the samples at each time point were clustered via partitioning around medoids (PAM) by using the “pam” function in the R library “cluster” [34]. The optimal number of clusters was chosen by maximizing the Calinski–Harabasz index [35], using “index.G1” function in the R library “clusterSim” [36], and the Silhouette index [37], using the “silhouette” function in the R library “cluster”. The result of clustering was visualized on a PCA plot, using the “s.class” function of the "ade4" package in R [38], and presented in Fig. 3. To determine genera that were differentially abundant between two enterotypes at each time point, LEfSe v1.0 [39] was used. The software uses the Kruskal-Wallis test to identify genera that are significantly different between two enterotypes at each time point, and used to build a Linear Discriminant Analysis (LDA) model, from which the relative difference between the two enterotypes is used to rank the genera. More details of LDA is fully described in [39].

The clustering analyses described above were repeated using the unrarefied microbiome data as suggested in [40], and the clustering results were compared with the results from using the rarefied data.

### Diversity analysis

The R package “vegan” [41] was used to investigate alpha diversity in this study. The diversity was measured using the Shannon index, computed here as $-\sum \limits_{i=1}^n{p}_i\ln \left({p}_i\right)$, where *p*_*i*_ was the proportional abundance of OTU *i*. A univariate linear regression model was formed to test the significance of fixed effects,

1 $$ {y}_{ijlkm}=\mu +{\mathrm{sex}}_i+{\mathrm{age}}_j\ast {\mathrm{family}}_l+{\mathrm{bs}}_k+{\mathrm{dl}}_m+{e}_{ijlkm}, $$

in which *μ* was the overall mean, age was the time point (weaning, week 15, off-test), family was the paternal half-sib family (*n* = 28), bs was the birth site (*n* = 3), dl was the dam line (*n* = 2) and potential interaction between the age and family, and a random residual effect *e*; the response was the Shannon index. The index at week 15 and off-test was pre-adjusted for contemporary group (*n* = 6). Fixed effects that were found insignificant from model (1) were removed from subsequent analyses. After testing the significance of the fixed effects, we explored longitudinal changes in the family effect using the model (1.1), which was analyzed using function “lmer” of the R package “lme4” [42].

1.1 $$ {y}_{ijlk}=\mu +{\mathrm{sex}}_j+{\mathrm{bs}}_k+{\mathrm{age}}_i{\mathrm{family}}_{il}+{e}_{ijlk} $$

We modified model (1) to form model (1.2) to test the impact of enterotypes on BF_18, BF_22, ADGw_14, and ADG14_22. Model (1.2) consisted of five fixed effects, including *days*, which were the age of the animal on the day when back fat thickness and live weight were measured; *enterotype* was the enterotype at weaning, week 15, or off-test; and *sex*, *family*, *bs*, and *e* remained the same as previously described.

1.2 $$ {y}_{jlkm}=\mu +{\mathrm{sex}}_j+\mathrm{days}+{\mathrm{family}}_l+{\mathrm{bs}}_k+{\mathrm{enterotype}}_m+{e}_{jlkm}, $$

Model (2) was formed to test the fixed effects of *sex*, *family*, and *bs*, as well as random permanent environmental effect of *litter* (*n* = 718), at three separate time points. The litter effect in this study refers to the nursing environment provided by the mother and siblings, influencing the development of individual pigs, potentially having profound impact on fitness and other phenotypic traits later in life. The random effects were assumed to be uncorrelated with each other. Covariance matrices of the random effects were equal to *I*${\sigma}_{\mathrm{litter}}^2$, *I*${\sigma}_e^2$, where *I* was an identity matrix.

2 $$ {y}_{jlkp}=\mu +{\mathrm{sex}}_j+{\mathrm{family}}_l+{\mathrm{bs}}_k+{\mathrm{litter}}_p+{e}_{jlkp} $$

Genetic parameters of the Shannon index were investigated using models (3), (4), and (5) as described below. The model included fixed effects of *sex* and *bs*. Random effects included *animal* and residual *e* in model (3). Model (4) was an extension of model (3) to include the permanent environmental effect *litter*. We estimated heritability of the Shannon index at each time point, as well as phenotypic and genetic correlations of the index among the three time points. Model (5) was an extension of model (4) to include the random effects of *pen* where the animals were raised after weaning. The response in model (5) was the Shannon index at week 15 and off-test. We estimated heritability of the index, as well as its phenotypic and genetic correlations between these two time points. Assumptions of the random effects of *litter* and *pen* remained similar to model (2). The random effect of *animal* in models (3), (4), and (5) was given a covariance matrix of ***A***${\sigma}_s^2$, in which ***A*** was the additive numerator relationship matrix, determined from a pedigree. The animal in models (4) and (5) was assumed uncorrelated with other random effects.

3 $$ {y}_{jks}=\mu +{\mathrm{sex}}_j+{\mathrm{bs}}_k+{\mathrm{animal}}_s+{e}_{jks} $$

4 $$ {y}_{jkps}=\mu +{\mathrm{sex}}_j+{\mathrm{bs}}_k+{\mathrm{litter}}_p+{\mathrm{animal}}_s+{e}_{jkps} $$

5 $$ {y}_{jknps}=\mu +{\mathrm{sex}}_j+{\mathrm{bs}}_k+{\mathrm{pen}}_n+{\mathrm{litter}}_p+{\mathrm{animal}}_s+{e}_{jknps} $$

Models (3), (4), and (5) differed from each other by the number of random effects. The goodness of fit of the models was evaluated via Likelihood Ratio Test (LRT). The *pen* effect applied to data collected only after weaning; thus, model (5) was used for data from week 15 and off-test only. Genetic parameters for OTU richness, which was the number of OTUs obtained in our rarefied data, were obtained in a similar manner. We also performed bivariate analyses for pairs of traits between Shannon index (at weaning and week 15, hereafter “Sha_w” and “Sha_15,” respectively) and BF_18, BF_22, ADGw_14, and ADG14_22. All of the linear models were tested using ASReml v.4.1 [43].

## Results

### Distribution of taxonomic abundance

**Fig. 1:**
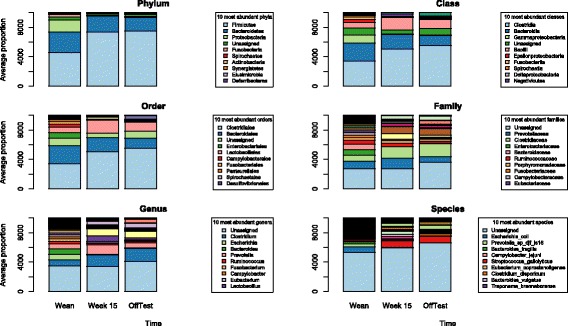
Distribution of abundance of microbiome taxa at various levels over weaning, week 15, and off-test. The *y*-axis is average proportion of relative abundance. The legend boxes list only the 10 most abundant taxanomic identity at each level

The results shown in Fig. 1 describe the abundance of microbial taxa at six different levels (phylum, class, order, family, genus, and species) at three different stages of pig development (weaning, 15 weeks of age, and off-test). There were 14, 21, 29, 54, 106, and 202 identified phyla, classes, orders, families, genera, and species, respectively. Details of the distributions are provided (see Additional file 4). Over the three time points, 95.79–97.80% of the OTUs were classified into six phyla: Firmicutes, Bacteroidetes, Proteobacteria, Fusobacteria, Spirochaetes, and Actinobacteria. Bacteria that were in the phylum Firmicutes represented the largest proportion of the total population followed by Bacteroidetes. These two phyla accounted for 73.61, 95.35, and 93.03% of all reads at weaning, week 15, and off-test, respectively. As the animals aged, the proportion of OTUs in the phylum Firmicutes increased, while the proportion of OTUs in the phylum Bacteroidetes decreased. At the phylum level, the proportion of OTUs that fell into the non-classified group was 3.86, 2.12, and 2.62% at weaning, week 15, and off-test, respectively.

Among the identified genera, two (*Ruminobacter* and *Akkermansia*) were unique to the weaners, 101 were present in all three age groups, one (*Anaerotruncus*) was present only in the weaners and the 15 weekers, one (*Cellulosilyticum*) was present only in the weaners and the off-test, and one (*Anaerostipes*) was present only in the 15 weekers and off-test animals. The number of genera that had significant difference in relative abundance counts (at least *P* < 0.01 with Bonferroni correction for multiple testing) between weaning and week 15 was 90, between weaning and off-test was 100, and between week 15 and off-test was 82. A full list of *P* values from Kruskal-Wallis tests at the genus level is provided in Additional file 5. *Clostridium* significantly increased in proportion over time, from 8.18 ± 6.68% (mean ± SD) at weaning to 15.50 ± 6.67 and 17.80 ± 4.93% at week 15 and off-test, respectively (*P* < 0.001 and *P* < 0.001, respectively). Other predominating genera at weaning included *Escherichia*, *Bacteroides*, and *Prevotella* with 7.73 ± 12.17, 7.30 ± 7.86, and 6.78 ± 5.76% of the total sequences, respectively. The average proportions of *Escherichia* and *Bacteroides* dropped significantly (*P* < 0.001) to 0.17 ± 0.87 and 0.15 ± 0.45% at week 15, and 0.23 ± 1.79 and 0.40 ± 0.87% at off-test, respectively, whereas the average proportion of *Prevotella* significantly increased to 13 ± 5.97% at week 15, then significantly (*P* < 0.001) dropped to 6.74 ± 3.08% at off-test.

At the species level, there were 202 identified species, of which 7 species (*Parabacteroides goldsteinii*, *Blautia glucerasea*, *Anaerotruncus sp. NML 070203*, *Anaerotruncus colihominis*, *Bacteroides nordii*, *Bacteroides caccae*, *Bacteroides eggerthii*) existed only at weaning and week 15, 4 species (*Clostridium methylpentosum*, *Ruminococcus albus*, *Bacteroides galacturonicus*, *Porphyromonas bennonis*) existed only in the week-15 and off-test individuals, 2 species (*Cellulosilyticum ruminicola*, *Collinsella stercoris*) were present only in the weaners and off-test animals, and 3 species (*Akkermansia muciniphila*, *Ruminobacter amylophilus*, *Alistipes putredinis*) were found exclusively in the weaners. Remarkable shifts in the abundance of sequences were observed in *E. coli* (7.66 ± 12.05, 0.17 ± 1.86, and 0.23 ± 1.78% at weaning, week 15, and off-test, respectively), *P. djf_ls16* (3.93 ± 3.94, 0.43 ± 0.60, and 0.13 ± 0.12%), *B. fragilis* (2.70 ± 6.00, 0.002 ± 0.03, and 0.06 ± 0.54%), *C. jejuni* (1.70 ± 4.01, 0.01 ± 0.13, and 0.01 ± 0.07%), *S. gallolyticus* (1.67 ± 3.90, 9.19 ± 5.85, and 8.64 ± 5.44%).

**Fig. 2:**
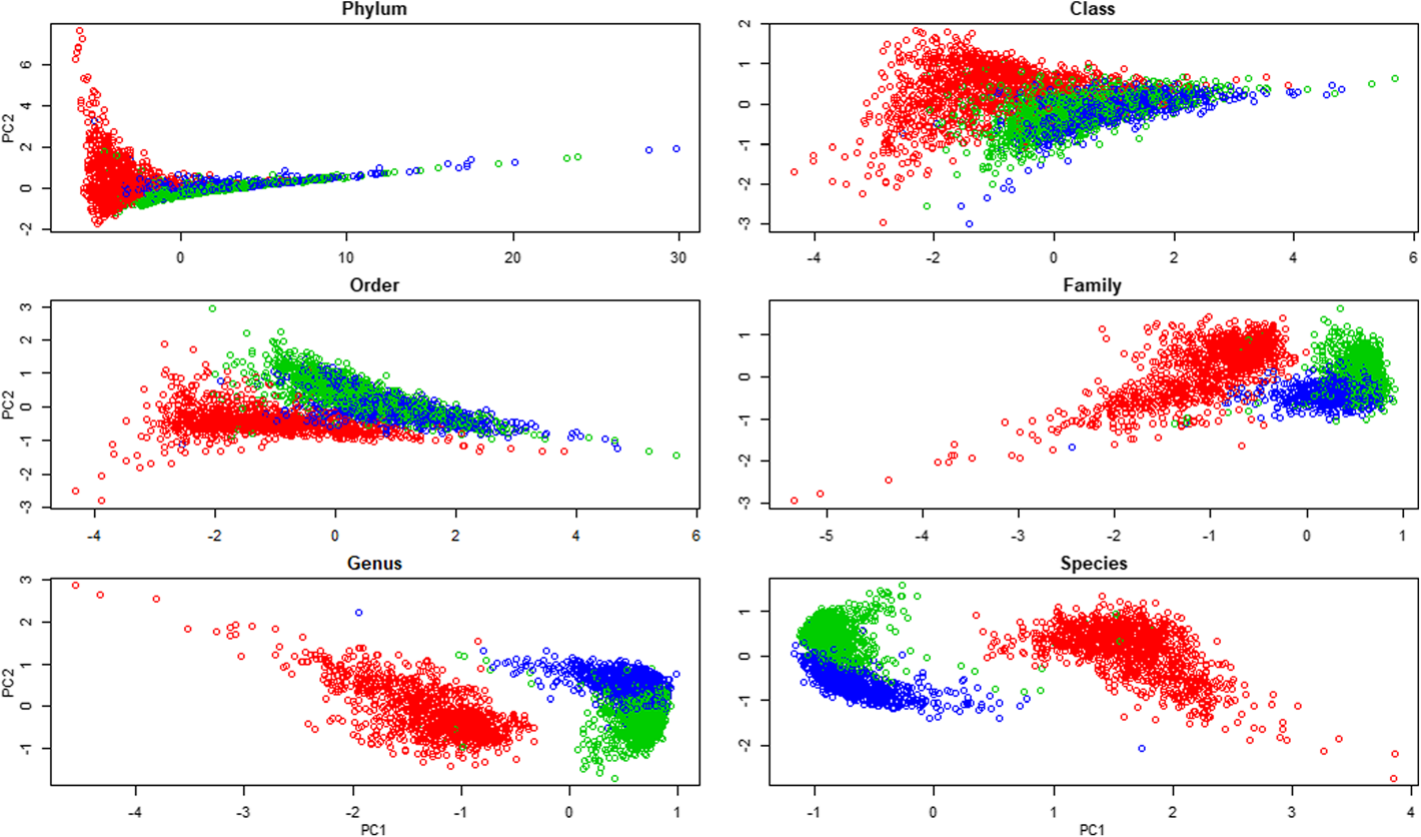
Scatter plots of samples at weaning (red circles), week 15 (green circles), and off-test (blue circles) by principal component 1 (PC1) and principal component 2 (PC2) at six taxonomic levels

Principal component analyses were carried out at the phylum, class, order, family, genus, and species levels. Scatter plots, based on the first two principal components (hereafter “PC1” and “PC2”), of all samples at the three time points are presented in Fig. 2. At each taxonomic level, PC1 mainly separated the weaners from the other two groups, whereas PC2 distinguished the 15-week olds from the off-test individuals. However, the effect of PC2 was clearly seen only in analyses at the family, genus, and species levels. Proportions of total variance explained by PC1 and PC2 are presented in Table 1. At the phylum level, PC1 and PC2 accumulatively accounted for 97.25% of the total variation. This proportion decreased to 75.04, 69.41, 44.38, 40.78, and 41.65% in analyses using the lower taxonomic levels, class, order, family, genus, and species, respectively.

**Table 1:** Proportion of variation explained by the first two principal components at different taxonomic levels and contribution of the top members to the first two principal components

Taxonomic level	PC1	PC2	Contribution to PC1	Contribution to PC2
Phylum	93.66	3.59		
Firmicutes			75.39	3.74
Proteobacteria			8.03	54.56
Bacteroidetes			6.31	26.99
Class	62.59	12.45		
Clostridia			45.98	3.35
Gammaproteobacteria			12.87	4.80
Fusobacteriia			7.91	11.20
Bacteroidia			7.61	22.58
Erysipelotrichia			4.86	3.43
Epsilonproteobacteria			4.51	1.19
Order	57.75	11.66		
Clostridiales			40.23	7.54
Enterobacteriales			11.00	14.12
Fusobacteriales			7.24	8.69
Bacteroidales			6.76	5.50
Pasteurellales			6.16	6.96
Erysipelotrichales			4.29	1.46
Campylobacterales			4.13	4.34
Fibrobacterales			2.42	2.91
Family	31.72	12.66		
Enterobacteriaceae			12.75	7.34
Bacteroidaceae			6.58	0.88
Fusobacteriaceae			6.27	4.74
Enterococcaceae			5.69	2.57
Pasteurellaceae			5.50	2.68
Peptostreptococcaceae			5.31	3.77
Clostridiaceae			4.52	7.89
Streptococcaceae			4.30	4.25
Campylobacteraceae			3.13	1.92
Prevotellaceae			3.05	9.18
Genus	30.56	10.22		
*Escherichia*			6.58	5.24
*Bacteroides*			3.84	0.34
*Fusobacterium*			3.76	1.45
*Peptostreptococcaceae*			3.47	1.98
*Enterococcus*			3.06	2.04
*Turicibacter*			2.85	3.04
*Clostridium*			2.77	4.36
*Streptococcus*			2.67	1.51
*Actinobacillus*			2.57	0.47
*Butyricimonas*			2.50	1.51
Species	33.49	8.16		
*Escherichia coli*			3.18	3.33
*Bacteroides fragilis*			1.90	1.13
*Streptococcus gallolyticus*			1.83	0.91
*Bacteroides vulgatus*			1.74	0.25
*Campylobacter jejuni*			1.60	0.71
*Turicibacter sanguinis*			1.54	1.95
*Clostridium scindens*			1.46	0.57
*Clostridium butyricum*			1.44	0.66
*Clostridium bolteae*			1.39	0.60
*Coprococcus catus*			1.38	0.11

*PC1 and PC2* are the two principal components 1 and 2, respectively

Table 1 also reveals that bacteria in the phylum Firmicutes were the main driver separating the weaners from the other two groups. They contributed to 75.39% of the variation in PC1. Two phyla, Proteobacteria and Bacteroidetes explained a total of 81.55% of PC2, which separated the 15-week olds from the off-test pigs. At the species level, PC1 was heavily loaded by ten species that distinguished the weaners from the other two groups, including *S. gallolyticus*, *T. sanguinis*, *C. butyricum*, *C. catus*, *E. coli*, *B. fragilis*, *B. vulgatus*, *C. jejuni*, *C. scindens*, and *C. bolteae*. PC2 was heavily loaded by the species that separated the 15 weekers from the off-test pigs, including *E. coli*, *T. sanguinis*, *L. amylovorus*, *O. sp. g2*, *P. sp. DJF b116*, *P. copri*, *P. sp. DJF ls16*, *P. sp. rs2*, *D. formicigenerans*, and *M. elsdenii*.

### Clustering pigs’ fecal microbiomes into enterotypes

**Fig. 3:**
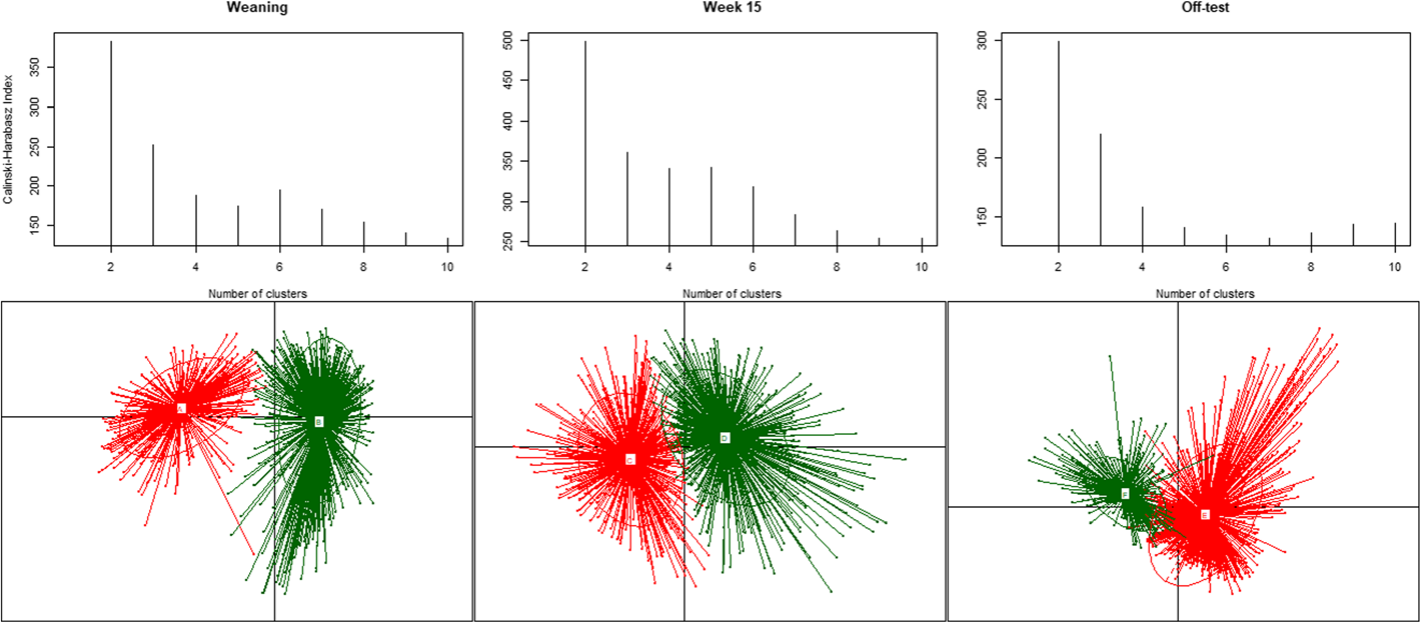
Calinski-Harabasz indexes (CH) for number of potential clusters of samples at weaning, week 15, and off-test. The highest CH value at each time point indicates optimal number of cluster/enterotypes. Samples at weaning formed two clusters, A and B. Samples at week 15 formed two clusters, C and D. Samples at off-test formed two clusters, E and F

The highest CH and RS were obtained for two clusters for both male and female samples collected at weaning, week 15, and off-test. We combined male and female samples at each time point and re-ran the CH test for optimal number of clusters. The CH indexes at each time point and clusters of samples corresponding to the optimal number of clusters are presented in Fig. 3. The optimal number of clusters for samples at each of the 3 time points was 2. We named them A and B at weaning, C and D at week 15, and E and F at off-test. The number of males and females in clusters A, B, C, D, E, and F were 166 and 226, 443 and 370, 276 and 291, 354 and 374, 399 and 438, and 219 and 217, respectively. The samples at each time point appeared to form 2 distinct clusters (Fig. 3).

**Fig. 4:**
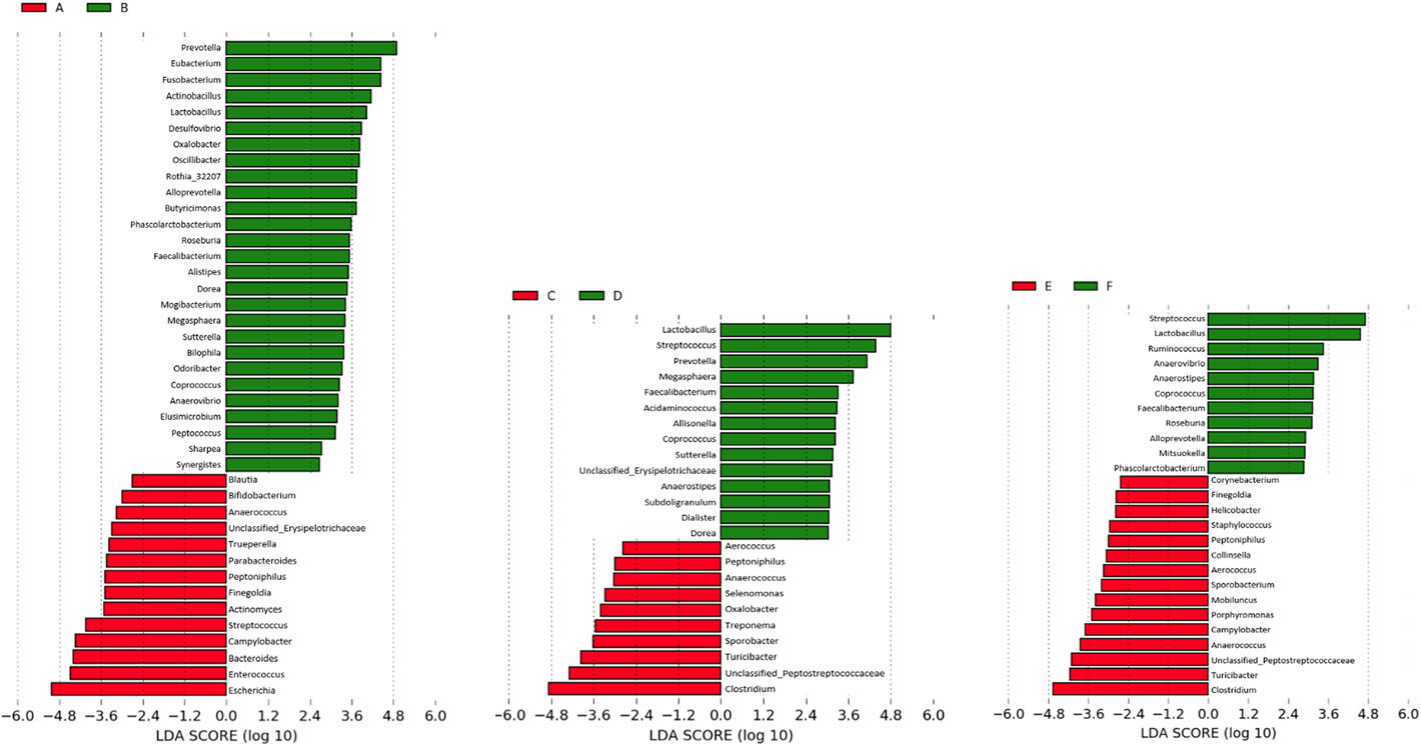
Effect size of genera that separate two enterotypes at weaning (A and B), week 15 (C and D), and off-test (E and F). The bar length represents a log_10_-transformed linear discriminant score. The colors represent which enterotype a genus is found to be more abundant compared to the other enterotype. Only absolute values of the effect size are considered when comparing one genus to another

Compositional characteristics of the enterotypes were studied, and genera that significantly (absolute LDA score > 2) separated one enterotype from the other at each time point are presented in Fig. 4. The overall observation was that at weaning type A and type B samples were significantly distinguished by 41 genera, of which 14 genera, led by *Escherichia*, were more abundant in type A samples than in type B samples, and 27 genera, led by *Prevotella*, were significantly more abundant in type B samples than in type A ones. Enterotypes C and D at week 15 and enterotypes E and F at off-test were significantly distinguished by 24 and 26 genera, respectively. Both type C and type E samples at week 15 and off-test, respectively, were significantly dominated by *Clostridium* and *Turicibater*, whereas both type D and type F samples at week 15 and off-test, respectively, were significantly enriched by *Lactobacillus* and *Streptococcus*.

Using model (1.2), we tested the potential impacts of enterotypes A, B, C, and D on BF_18, BF_22, ADGw_14, and ADG14_22, as well as the effect of enterotypes E and F on BF_22 and ADG14_22. Enterotypes A and B did not have significant effect on the traits (all *P* > 0.05). Enterotypes C and D had significant effect on BF_18 (*P* < 0.001) and BF_22 (*P* < 0.001) but not on ADGw_14 and ADG14_22 (both *P* > 0.05). Enterotypes E and F had significant effect on BF_22 but not on ADG14_22. Enterotypes D and F were both significantly enriched mainly with *Lactobacillus*, *Streptococcus*, and *Prevotella* compared to *Clostridium*-enriched enterotypes C and E, and our analysis revealed that enterotype D at week 15 was associated with an increase of 0.08 and 0.10 cm in backfat thickness at week 18 (BF_18) and week 22 (BF_22), respectively, compared to enterotype C. Similarly, enterotype F was associated with an increase of 0.10 cm in BF_22 compared to enterotype E.

A subset of 1039 animals that had samples collected at weaning, week 15, and off-test was used to evaluate the frequency with which individual animals transitioned between enterotypes as they developed. The percentage of type A weaners developing into types C and D at week 15 was 43.11 and 56.89%, respectively. For type B weaners, 41.26 and 58.74% of them became type C and D, respectively. From weaning, 32.55 and 32.66% of type A and type B pigs, respectively, grew to type F pigs at off-test. From week 15, 22.07 and 40.23% of type C and type D pigs, respectively, joined the type F group at off-test. A detailed breakdown of the number of samples in each enterotype is provided (see Additional file 6).

We tested whether or not the distribution of samples in the enterotypes (see Additional file 7) was affected by the family factor, using contingency tables and a Chi-squared test in R. Sire families had significant impact on the distribution of animals into enterotypes at weaning (*P* < 0.005), week 15 (*P* < 0.05), and off-test (*P* < 0.001).

### Longitudinal analysis of microbiome diversity

**Fig. 5:**
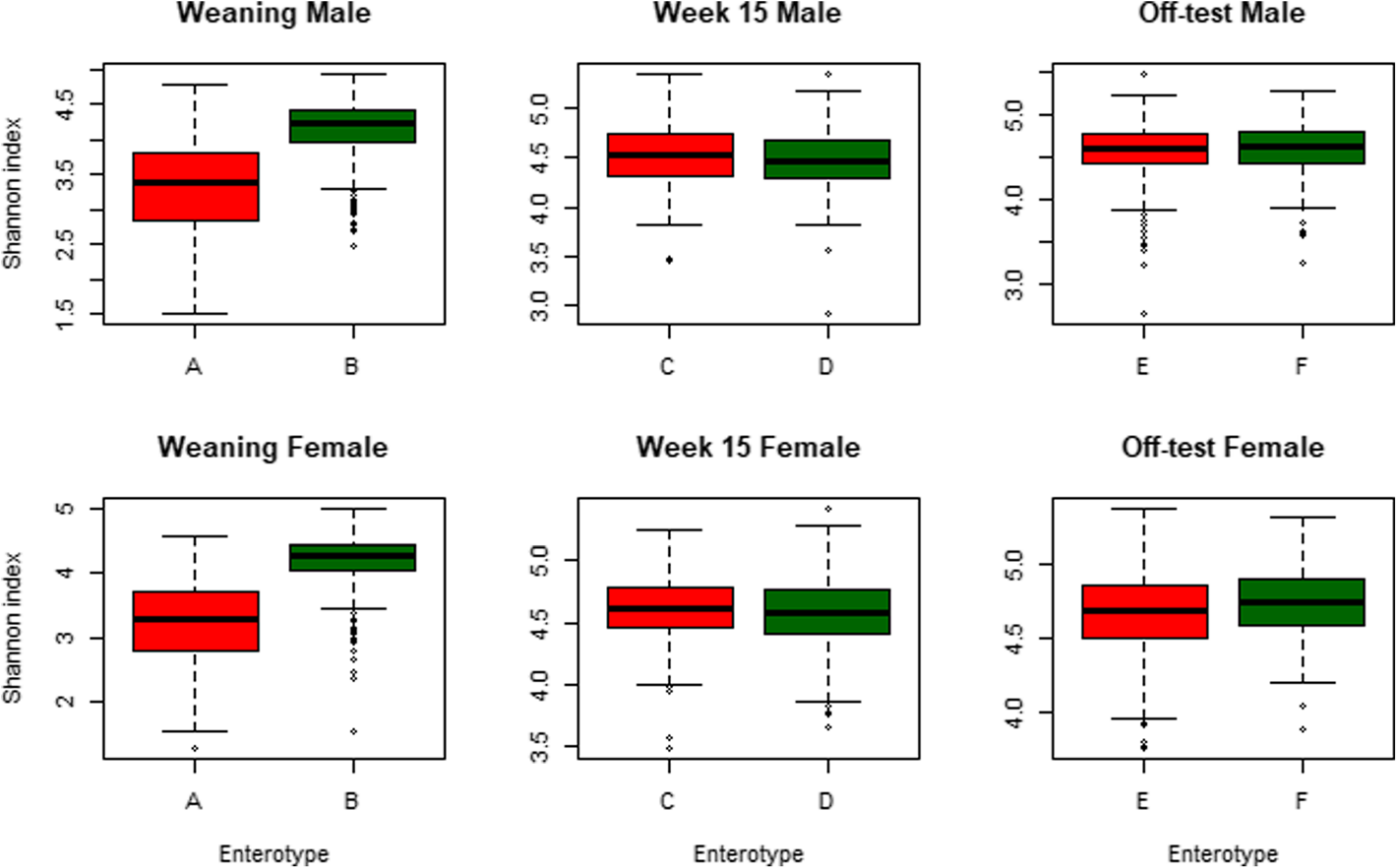
Box plots of the Shannon index in each enterotype by sex at weaning, week 15, and off-test

Alpha diversity of the microbiome was evaluated using the Shannon index and plotted in Fig. 5 by sex and time point. At weaning, the average Shannon indices for type A and type B weaners was 3.33 ± 0.67 and 4.13 ± 0.43 in the males and 3.21 ± 0.68 and 4.18 ± 0.43 in the females, respectively. Results from *t* tests showed that those means were significantly different between types A and B in both male and female weaners (*P* < 0.001). At week 15, types C and D had an average Shannon indices of 4.50 ± 0.30 and 4.47 ± 0.29 in males and 4.60 ± 0.26 and 4.57 ± 0.27 in females, respectively. These averages were not significantly different between the two enterotypes in both sex groups (*P* > 0.05). The average Shannon indices for type E and type F off-test pigs were 4.57 ± 0.32 and 4.59 ± 0.31 in males and 4.67 ± 0.28 and 4.73 ± 0.23 in the females, respectively. Significant difference in the Shannon index between type E and type F animals was observed only in the female pigs (*P* < 0.01).

The test from model (1) showed a significant impact of sex (*P* < 0.05), bs (*P* < 0.001), and interaction between age and family (*P* < 0.001) on the alpha diversity of microbiome measured using the Shannon index. The estimated effects of the fixed factors are listed in Additional file 8. The effect of *dl* was not significant (*P* > 0.05) and was thus removed in subsequent analyses. We compared the clustering results from the rarefied data to the results from the unrarefied, and found no major differences in the clusters and enterotypes. For the reader reference, we put the results using the unrarefied data in Additional file 9, Additional file 10, and Additional file 11.

**Fig. 6:**
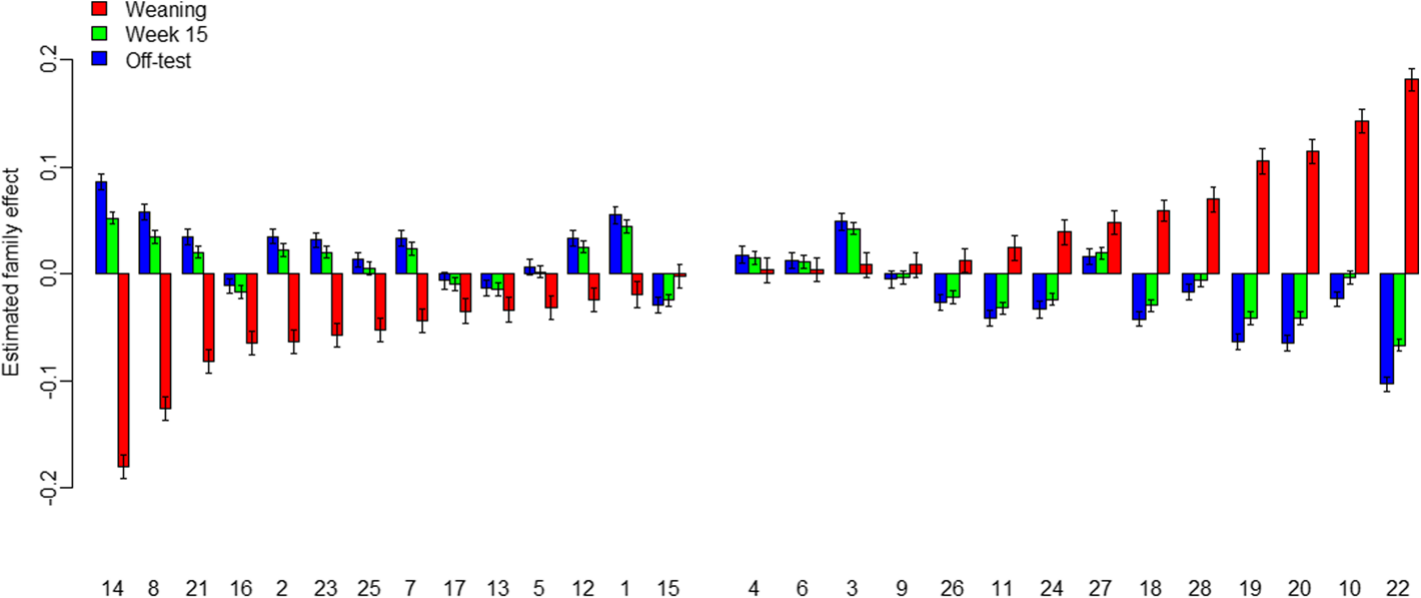
Family effect on the Shannon index estimated for each family at weaning, week 15, and off-test

Longitudinal changes in random effects of family were estimated using model (1.1) and are plotted in Fig. 6, which shows a group of 14 families (1, 2, 5, 7, 8, 12, 13, 14, 15, 16, 17, 21, 23, 25) with negative estimated effect on the Shannon index at weaning, indicating their diversity was below the average of the 28 families investigated. The other 14 (3, 4, 6, 9, 10, 11, 18, 19, 20, 22, 24, 26, 27, 28) families had their estimated family effect above the population mean. The latter group appears to show a trend, though not consistently across the 14 families, where families that had very high diversity at weaning tended to have very low diversity at week 15 and off-test. This tendency is even less consistent in the other group of 14 families. Pearson’s correlation for the estimated family effect on the diversity between weaning and week 15 was − 0.70, between week 15 and off-test was − 0.82, and between week 15 and off-test was 0.98.

Analyses performed at individual time points and described in model (2) revealed significant impact of *sex* (*P* < 0.05) and *bs* (*P* < 0.001) and insignificant effect of *family* (*P* > 0.05) at weaning. At week 15 and off-test, both *family* and *sex* effects were significant (*P* < 0.001), whereas the *bs* effect was insignificant (*P* > 0.05).

We considered the index as a phenotypic record and hence attempted to estimate its genetic parameters. The results from model (3), not including the litter effect, are presented in Table 2, suggesting that the measure was lowly heritable at weaning and week 15 (0.04 ± 0.04 and 0.15 ± 0.06, respectively) but moderately heritable at off-test (0.33 ± 0.10). Very weak negative phenotypic correlation was detected between weaning and week 15 (− 0.01 ± 0.03), as well as between weaning and off-test (− 0.04 ± 0.03). Considering the standard error, those correlations were almost zero. However, the measures at week 15 were positively correlated with those at off-test phenotypically, 0.15 ± 0.03. Genetically, the index at weaning was negatively correlated with those at week 15 and off-test, respectively − 0.17 ± 0.48 and − 0.34 ± 0.47. However, the genetic correlation for this index between week 15 and off-test was 0.44 ± 0.25.

**Table 2:** Estimated genetic parameters of the Shannon index and their standard errors

	Weaning	Week 15	Off-test
Weaning	0.04 ± 0.04	− 0.01 ± 0.03	− 0.04 ± 0.03
Week 15	− 0.17 ± 0.48	0.15 ± 0.06	0.15 ± 0.03
Off-test	− 0.34 ± 0.47	0.44 ± 0.25	0.33 ± 0.10

Model not including litter effect. Values on the diagonal are heritability, above the diagonal are phenotypic correlations, and below the diagonal are genetic correlations

We also estimated genetic parameters of OTU richness and observed a trend similar to the one found with the Shannon index. Table 3 shows almost zero heritability, 0.03 ± 0.04, for the OTU richness at weaning, but moderate heritabilities of 0.26 ± 0.08 and 0.24 ± 0.08 at week 15 and off-test, respectively. A very weak genetic correlation was estimated between weaning and week 15 (0.07 ± 0.52), whereas the estimated genetic correlations between weaning and off-test, as well as between week 15 and off-test, were 0.25 ± 0.53 and 0.11 ± 0.25, respectively. Very large standard errors were observed for the genetic correlations between weaning and week 15, as well as between weaning and off-test for the Shannon index and OTU richness.

**Table 3:** Estimated genetic parameters of the OTU richness and their standard errors

	Weaning	Week 15	Off-test
Weaning	0.03 ± 0.04	0.00 ± 0.03	− 0.02 ± 0.03
Week 15	0.07 ± 0.52	0.26 ± 0.08	0.07 ± 0.03
Off-test	0.25 ± 0.53	0.11 ± 0.25	0.24 ± 0.08

Model not including litter effect. Values on the diagonal are heritability, above the diagonal are phenotypic correlations, and below the diagonal are genetic correlations

Tables 4 and 5 show genetic parameters of the Shannon index and OTU richness estimated using model (4). Heritabilities of the Shannon index and OTU richness at weaning were 0.02 ± 0.04 and 0.01 ± 0.03, respectively. It should be noted that small estimated additive genetic variances at weaning might have inflated the estimated covariances between weaning and the other two time points. The estimated heritabilities of the Shannon index and OTU richness slightly changed to 0.16 ± 0.07 and 0.24 ± 0.09, respectively, at week 15, whereas they decreased to 0.22 ± 0.09 and 0.20 ± 0.08, respectively, at off-test. Interestingly, the genetic correlation between week 15 and off-test for the Shannon index and OTU richness increased remarkably to 0.65 ± 0.23 and 0.39 ± 0.27, respectively.

**Table 4:** Estimated genetic parameters of the Shannon index and their standard errors

	Weaning	Week 15	Off-test
Weaning	0.02 ± 0.04	− 0.01 ± 0.03	− 0.04 ± 0.03
Week 15	− 0.23 ± 0.49	0.16 ± 0.07	0.16 ± 0.03
Off-test	− 0.96 ± 0.43	0.65 ± 0.23	0.22 ± 0.09

Model including litter effect. Values on the diagonal are heritability, above the diagonal are phenotypic correlations, and below the diagonal are genetic correlations

**Table 5:** Estimated genetic parameters of the OTU richness and their standard errors

	Weaning	Week 15	Off-test
Weaning	0.01 ± 0.03	0.00 ± 0.03	− 0.03 ± 0.03
Week 15	–	0.24 ± 0.09	0.08 ± 0.03
Off-test	–	0.39 ± 0.27	0.20 ± 0.08

Model including litter effect. Values on the diagonal are heritability, above the diagonal are phenotypic correlations, and below the diagonal are genetic correlations. Genetic correlations between weaning and week 15, as well as between weaning and off-test, were not estimable

Tables 6 and 7 present genetic parameters of the Shannon index and OTU richness estimated using model (5). Adding the random effect of pen in model (5) accounted for the immediate environment shared among pen mates at week 15 and off-test. This led heritability estimates for the Shannon index to decrease slightly to 0.17 ± 0.08 and 0.19 ± 0.09 at week 15 and off-test, respectively; heritability estimates for OTU richness also decreased slightly to 0.23 ± 0.09 and 0.17 ± 0.08 at week 15 and off-test, respectively, when compared to the estimates from model (4). The genetic correlation for the two traits between week 15 and off-test increased to 0.80 ± 0.24 and 0.52 ± 0.29, respectively. Our results might suggest there is very little or no influence of the host’s genetics on gut microbiome diversity at weaning, when the gut microbiota may be significantly influenced by environmental factors coincident with the weaning process.

**Table 6:** Estimated genetic parameters of the Shannon index and their standard errors

	Weaning	Week 15	Off-test
Weaning	–	–	–
Week 15	–	0.17 ± 0.08	0.16 ± 0.03
Off-test	–	0.80 ± 0.24	0.19 ± 0.09

Model including litter effect and pen effect. Values on the diagonal are heritability, above the diagonal are phenotypic correlations, and below the diagonal are genetic correlations. The pen effect did not apply to weaning samples; thus, model (5) was used for only week 15 and off-test

**Table 7:** Estimated genetic parameters of the OTU richness and their standard errors

	Weaning	Week 15	Off-test
Weaning	–	–	–
Week 15	–	0.23 ± 0.09	0.08 ± 0.03
Off-test	–	0.52 ± 0.29	0.17 ± 0.08

Model including litter effect and pen effect. Values on the diagonal are heritability, above the diagonal are phenotypic correlations, and below the diagonal are genetic correlations. The pen effect did not apply to weaning samples; thus, model (5) was used for only week 15 and off-test

The LRT revealed significant improvement in goodness of fit when including the litter effect, model (3) vs model (4) (*P* < 0.001), as well as when including the pen effect, model (4) vs model (5) (*P* < 0.001), for both Shannon index and OUT richness. The results suggest that it is important to include both litter and pen in the model when estimating genetic parameters for the two traits.

Relationships between Sha_w and Sha_15 with BF_18, BF_22, ADGw_14, and ADG14_22 are provided in Table 8. The phenotypic correlations between the Sha_w and BF_18, BF_22, ADGw_14, and ADG14_22 were all positive, between 0.03 and 0.06, whereas the correlations between Sha_15 and those four traits were all negative, between − 0.10 and − 0.08. Genetic correlations between BF_18, BF_22, ADGw_14, and ADG14_22 and Sha_w were 0.38 ± 0.43, 0.55 ± 0.41, − 0.73 ± 0.51, and 0.44 ± 0.48, respectively, whereas their correlations with Sha_15 were − 0.53 ± 0.23, − 0.45 ± 0.25, − 0.53 ± 0.32, and − 0.53 ± 0.29, respectively. The genetic correlations between Sha_15 and the four traits were all strong and more consistent than the estimates between those four traits with Sha_w, which had very large standard errors. This result might suggest that Sha_15 in our data could be a better predictor of BF18, BF22, ADGw_14, and ADG14_22 than Sha_w.

**Table 8:** Heritability and phenotypic/genetic correlations between back fat, average daily gain, and Shannon index at weaning and week 15

	Sha_w	Sha_15	BF_18	BF_22	ADGw_14	ADG14_22
Sha_w	0.04 ± 0.04	–	0.05 ± 0.03	0.06 ± 0.03	0.04 ± 0.03	0.07 ± 0.03
Sha_15	–	0.18 ± 0.08	− 0.10 ± 0.03	− 0.08 ± 0.03	− 0.09 ± 0.03	− 0.09 ± 0.03
BF_18	0.42 ± 0.50	− 0.53 ± 0.23	0.30 ± 0.11	–	0.43 ± 0.03	0.31 ± 0.03
BF_22	0.52 ± 0.49	− 0.45 ± 0.25	–	0.28 ± 0.10	–	0.45 ± 0.03
ADGw_14	− 0.73 ± 0.51	− 0.53 ± 0.32	0.29 ± 0.32	–	0.09 ± 0.06	–
ADG14_22	0.44 ± 0.48	− 0.53 ± 0.29	0.10 ± 0.29	0.24 ± 0.28	–	0.17 ± 0.08

Values on the diagonal are heritability, above the diagonal are phenotypic correlations, and below the diagonal are genetic correlations. *Sha_w* Shannon index at weaning, *Sha_15* Shannon index at week 15, *BF_18* back fat thickness at week 18, *BF_22* back fat thickness at week 22, *ADGw_14* average daily gain from weaning to week 14, *ADG14_22* average daily gain from week 14 to week 22

## Discussion

The data used in this project were collected from a population of crossbred pigs whose fecal bacterial communities were sampled at weaning, week 15, and off-test. The overall goal of this project was to investigate the potential contribution of information from the pig fecal microbiome to the genetic improvement of the pig. The analyses presented here are our first steps toward better understanding temporal changes in the pig’s fecal microbiome, with respect to both community composition and diversity, and toward exploring the potential influence of the host’s genetic background on variation in microbiota diversity over time.

The gut microbiota of the animals in this study were predominated by two phyla, Firmicutes and Bacteroidetes, in agreement with published research [6, 8, 44–46]. However, the most abundant genus at all three time points in our dataset was *Clostridium*, instead of *Prevotella* as reported in [8, 46, 47]. The colonization of *Clostridium* and other genera, including *Escherichia* and *Prevotella*, begins immediately following birth [48, 49] and could be disrupted by changes in living environment and the host conditions [50, 51]. At weaning, the pigs were removed from their mothers and exposed to changes in both diet and living environment. All of these changes might have impacted the gut microbial ecosystem established prior to weaning, during which the piglets were trained on concentrate food. Our data suggest weaning animals can be divided into two distinct enterotypes: (1) a *Prevotella*-enriched cluster which might represent those communities accustomed to feed rich in plant polysaccharides and (2) an *Escherichia*-enriched cluster in which the presence of *Enterococcus* might indicate gut health disruption [52].

Analyses of enterotypes in this study were based on the assumption that there existed at least two enterotypes among the pigs at each time point, and we were interested in their potential association with back fat deposition and growth rate. The pigs used in this study did not clustered into *Prevotella* and *Ruminococcus* enterotypes as reported in pigs [2] nor did they group distinctively into *Prevotella*, *Bacteroides*, and *Ruminococcus* enterotypes as reported in human research [33, 53]. The difference in enterotypes between this research and aforementioned studies might have been partly attributed to the difference in the genetic background of the host. From a genetics point of view, the pigs used in this study were from a population under selection for growth and thus might have been less diverse than the hosts in the other studies. In terms of association between enterotypes and phenotypes, the results presented herein contradicted the findings reported in [53], in which significant association was observed between enterotypes at 36 days of age with average daily gain at 70 days of age. Enterotypes identified among week 15 and off-test pigs in our study were significantly associated with only back fat thickness. Association between the identified enterotypes and alpha diversity was not clear in our study and might be further studied by investigating the genera underlying differences among the enterotypes.

A highly diverse microbiota is beneficial to the host [54–56]. We have demonstrated that alpha diversity in our data was under significant influence of family strata and have identified families whose progeny had increasing microbiota diversity through week 15 and off-test. We used paternal half-sib families in this research, thus each family represented a breeding male pig that was mated to several female pigs to produce the offspring. The significant variation in alpha diversity we observed among the families in this study suggests bacterial biodiversity within the pig gut might be influenced by the host’s genetics. The diversity index used in this study, to the best of our knowledge, has never been reported in the current literature as a trait. In animal production, there are index traits that are computed based on actual measures on animals, such as feed conversion ratio (the ratio of the weight of feed consumed by an animal to its body weight gain over the same period of time) and residual feed intake (which is modeled from feed intake, weight gain, and fat thickness). In humans, body fat deposition has been associated with alpha diversity of the gut microbiota [57–59]. Disease conditions have also been correlated with decreases in microbiome diversity [60–64]. Despite numerous studies linking the gut microbiota’s composition and diversity to host health conditions, the current literature has no reports on genetic parameters of the diversity of the gut microbiome.

Before this discussion extends to the genetic parameters of microbiota diversity, it might be worth clarifying the use of permanent environmental effect of litter in the statistical models that we used in this paper. The permanent environmental effect in this study refers to the nursing environment provided by the mother and siblings. This environment influences the development of individual pigs and potentially has a profound impact on fitness and other phenotypic traits later in life [65–67]. It is thought that immediately after birth, newborn pigs begin acquiring their gut microbiota from a combination of environmental exposures and vertical transmission of maternal microbes [68, 69]. It has been shown that maternal diet and antibiotic exposures may induce long lasting impacts on gut microbiota establishment, gut biology, and the growth performance of progeny [3, 70, 71].

This study is the first to describe OTU richness and alpha diversity as phenotypic traits in farm animals and the first to estimate their genetic parameters at three key stages of pig development. The heritability of 0.15 – 0.33 reported in this study means that 15 – 33% of the variation in alpha diversity measured in our pigs, at week 15 and off-test, was due to genetics. Examples of traits with similar heritability range include residual feed intake (*h*^2^ = 0.13) and belly weight (*h*^2^ = 0.28) [72]; tenderness (*h*^2^ = 0.26), meat color (*h*^2^ = 0.28), growth rate (*h*^2^ = 0.30), and feed conversion ratio (*h*^2^ = 0.29) [73–75]. These traits have been targeted for selection in pig breeding programs around the world due to their economic importance to the pork industry. Alpha diversity, reported to be associated with gut health, and found in this study to be strongly correlated with back fat thickness and average daily gain, which are the two important components of feed efficiency in livestock production, could very well be an indicator trait for genetic selection. Our results also suggest that diversity at weaning might not be an accurate predictor of diversity at later stages in life. If alpha diversity is to be used as an indicator trait, its availability soon after weaning will be beneficial to a selection process. Therefore, we suggest further investigation into alpha diversity at time points earlier than week 15.

Domestic pigs are similar to humans in terms of anatomy, genetics, and physiology [17]. They can be used as a model to study human diseases due to their similar clinical manifestations and susceptibility to many enteric pathogens that afflict humans [18–22]. Furthermore, outbred pigs, like the ones used in the present study, best mimic animal variation reflective of outbred human populations [16]. This study estimates the contribution of host genetics to the diversity of pig’s gut microbiota. Taking this finding into human studies, a better understanding of the relationship between host genetics and microbiome diversity might lead to changes in research direction to improve the human gut health.

## Conclusions

This study was conducted on a group of crossbred pigs living through three stages of life (weaning, week 15, and off-test) and was designed to explore longitudinal changes in fecal microbiome composition and diversity, as well as the influence of host genetics on microbiome diversity. Two enterotypes were identified at each stage of life, but only enterotypes at week 15 and off-test were proven to be associated with back fat thickness. Microbiome alpha diversity as measured using the Shannon index was found to be lowly to moderately heritable at week 15 and off-test. The diversity at these two time points was also found to have strong genetic correlation to each other. The diversity index at week 15 was also strongly correlated with back fat and average daily gain of the pigs. These findings may lead to a new direction of research in animal breeding and genetics and suggest potentially significant utility for gut microbiome data in the genetic evaluation process.

## Additional files

Additional file 1: Table S1. Diet formulae and their nutritional values. (PDF 70 kb)

Additional file 2: Table S2. Vaccinations. **Table S3.** Injectable medications. **Table S4.** Water medications. (PDF 36 kb)

Additional file 3: Table S5. Distribution of samples across families, sex, and time points. (PDF 39 kb)

Additional file 4: Table S6. Proportion of OTU counts at weaning, week 15, and off-test by phylum. **Table S7.** Proportion of OTU counts at weaning, week 15, and off-test by class. **Table S8.** Proportion of OTU counts at weaning, week 15, and off-test by order. **Table S9.** Proportion of OTU counts at weaning, week 15, and off-test by family. **Table S10.** Proportion of OTU counts at weaning, week 15, and off-test by genus. **Table S11.** Proportion of OTU counts at weaning, week 15, and off-test by species. (PDF 141 kb)

Additional file 5: Table S12. *P* values from Kruskal-Wallis tests for differences in abundance between pairs of time points at the genus level. (PDF 71 kb)

Additional file 6: Figure S1. Sample flow between enterotypes through weaning, week 15, and off-test. (PDF 46 kb)

Additional file 7: Table S13. Distribution of samples in enterotypes by family. (PDF 43 kb)

Additional file 8: Table S14. Effects and standard errors of family and time on alpha diversity estimated from model (1). (PDF 19 kb)

Additional file 9: Table S15. Proportion of variation explained by the first two principal components at different taxonomic levels and contribution of top members to the first two principal components, using unrarefied microbiome data. (PDF 47 kb)

Additional file 10: Figure S2. Scatter plots of samples by principal component 1 (PC1) and principal component 2 (PC2) at six taxonomic levels, using unrarefied microbiome data. (PDF 447 kb)

Additional file 11: Figure S3. Calinski-Harabasz indexes (CH) for number of potential clusters of samples at weaning, week 15, and off-test, using unrarefied microbiome data. (PDF 174 kb)

## Abbreviations

ADG14_22: Average daily body weight gain from week 14 to 22 of the experiment
ADGw_14: Average daily body weight gain from weaning to week 14 of the experiment
BF_18: Back fat thickness at week 18 of the experiment
BF_22: Back fat thickness at week 22 of the experiment
Bs: Birth site
Cg: Contemporary group
Dl: Dam line
OTU: Operational taxonomic unit
PC1: First principal component
PC2: Second principal component
Sha_15: Shannon index at week 15 post-weaning
Sha_w: Shannon index at weaning
SNP: Single nucleotide polymorphism
