# Supplementary material for: Host contributes to longitudinal diversity of fecal microbiota in swine selected for lean growth
Source: Microbiome. 2018 Jan 4;6:4. doi: 10.1186/s40168-017-0384-1 (PMC5755158; doi:10.1186/s40168-017-0384-1)
Supplement: Supplementary file 1 — Diet formulae and their nutritional values. (PDF 70 kb) [file 40168_2017_384_MOESM1_ESM.pdf]

Table S1. Diet formulae and their nutritional values

		Nursery 3	Nursery 4	GF-1	GF-2		GF-3		GF-4		GF-5		GF-6		GF-7
Ingredient		Barrow/Gilt	Barrow/Gilt	Barrow/Gilt	Barrow	Gilt	Barrow	Gilt	Barrow	Gilt	Barrow	Gilt	Barrow	Gilt	Barrow/Gilt
Corn		660.60	861.88	800.62	1020.46	1013.36	1236.71	1204.82	1382.01	1335.94	1481.33	1435.26	1530.99	1499.03	1534.50
Corn germ meal		48.01	341.84	564.17	464.72	467.93	366.89	381.32	301.16	322.00	256.23	277.07	233.76	248.22	232.18
Soybean meal		326.70	594.00	490.79	394.42	397.53	299.63	313.60	235.93	256.12	192.39	212.59	170.62	184.63	169.09
Fat - yellow grease (post-pellet)				64.46	47.08	47.64	29.99	32.51	18.50	22.14	10.65	14.29	6.72	9.25	6.45
Limestone			30.20	27.99	25.81	25.88	23.66	23.97	22.21	22.67	21.23	21.69	20.73	21.05	20.70
Pelleting aid				10.00	10.00	10.00	10.00	10.00	10.00	10.00	10.00	10.00	10.00	10.00	10.00
L-Lysine HCl (98%)			8.91	9.65	8.17	8.22	6.71	6.93	5.74	6.05	5.07	5.38	4.73	4.95	4.71
Salt			11.17	9.14	9.13	9.13	9.12	9.13	9.12	9.12	9.11	9.12	9.11	9.11	9.11
Fat - yellow grease		41.55	13.86	7.00	7.00	7.00	7.00	7.00	7.00	7.00	7.00	7.00	7.00	7.00	7.00
Monocalcium phosphate (21%)			18.13	5.51	4.52	4.55	3.54	3.69	2.89	3.09	2.44	2.65	2.21	2.36	2.20
HMTBa			2.26	4.50	3.29	3.33	2.09	2.27	1.29	1.55	0.74	1.00	0.47	0.65	0.45
L-Threonine (98%)			2.07	2.35	1.78	1.80	1.23	1.31	0.85	0.97	0.60	0.72	0.47	0.55	0.46
Trace mineral premix			1.98	2.00	1.87	1.87	1.73	1.75	1.64	1.67	1.58	1.61	1.55	1.57	1.55
Phytase 2500			1.90	0.80	0.76	0.76	0.73	0.73	0.70	0.71	0.68	0.69	0.67	0.68	0.67
Vitamin premix			0.99	0.60	0.57	0.57	0.55	0.55	0.53	0.53	0.52	0.52	0.51	0.51	0.51
Copper chloride (58%)			0.68	0.43	0.43	0.43	0.43	0.43	0.43	0.43	0.43	0.43	0.43	0.43	0.43
Nursery basemix		791.99													
DDGS		111.15	76.52												
Mecadox 2.5 (g/lb)		20	20												
Zinc oxide (72%)			6.93												
Organic acidifier			5.94												
Carbohydrase			0.74												
Total:		2000	2000	2000	2000	2000	2000	2000	2000	2000	2000	2000	2000	2000	2000
Nutrient	Units														
Metabolizable energy	Kcal/lb	1500	1519.371	1460.004	1460.18	1460.17	1460.35	1460.33	1460.47	1460.43	1460.55	1460.51	1460.59	1460.56	1460.59
Crude protein	%	20.103	22.561	21.281	18.59	18.68	15.95	16.34	14.17	14.74	12.96	13.52	12.35	12.74	12.31
Cystine, Dig	%	0.293	0.272	0.244	0.22	0.22	0.20	0.20	0.19	0.19	0.18	0.18	0.17	0.18	0.17
Isoleucine, Dig	%	0.675	0.786	0.715	0.62	0.62	0.52	0.53	0.45	0.47	0.41	0.43	0.39	0.40	0.38
Lysine, Total	%	1.405	1.521	1.454	1.23	1.24	1.01	1.05	0.87	0.91	0.77	0.81	0.72	0.75	0.71
Lysine, Dig	%	1.25	1.34	1.27	1.07	1.08	0.87	0.90	0.74	0.78	0.65	0.69	0.61	0.64	0.60
Leucine, Dig	%	1.469	1.586	1.439	1.31	1.31	1.18	1.20	1.09	1.12	1.03	1.06	1.00	1.02	1.00
Met + Cys, Dig	%	0.707	0.765	0.725	0.62	0.62	0.51	0.53	0.44	0.47	0.40	0.42	0.37	0.39	0.37
Threonine, Dig	%	0.76	0.804	0.762	0.65	0.65	0.54	0.55	0.46	0.48	0.41	0.43	0.38	0.40	0.38
Tryptophan, Dig	%	0.223	0.232	0.216	0.18	0.18	0.15	0.16	0.13	0.14	0.12	0.12	0.11	0.11	0.11
Valine, Dig	%	0.826	0.871	0.826	0.72	0.72	0.62	0.63	0.55	0.57	0.50	0.52	0.47	0.49	0.47
Phosphorus	%	0.728	0.689	0.557	0.50	0.50	0.44	0.45	0.41	0.42	0.38	0.39	0.37	0.38	0.37
P, Available	%	0.569	0.4	0.3	0.27	0.27	0.24	0.24	0.21	0.22	0.20	0.21	0.19	0.20	0.19
Calcium	%	0.809	0.896	0.7	0.63	0.63	0.57	0.58	0.52	0.54	0.49	0.51	0.48	0.49	0.48
Moisture	%	13.735	13.035	13.172	13.60	13.58	14.01	13.95	14.29	14.20	14.48	14.39	14.58	14.52	14.58
Crude fat	%	5.476	3.03	5.458	4.82	4.84	4.18	4.28	3.76	3.89	3.47	3.60	3.32	3.42	3.31
Crude fiber	%	2.117	3.18	3.486	3.17	3.18	2.85	2.90	2.64	2.71	2.50	2.57	2.43	2.47	2.42
ADF	%	2.92	5.01	5.367	4.86	4.88	4.37	4.44	4.03	4.14	3.81	3.91	3.69	3.76	3.68
NDF	%	6.507	12.75	15	13.58	13.63	12.19	12.40	11.26	11.55	10.62	10.91	10.30	10.50	10.28
