# Supplementary material for: Host contributes to longitudinal diversity of fecal microbiota in swine selected for lean growth
Source: Microbiome. 2018 Jan 4;6:4. doi: 10.1186/s40168-017-0384-1 (PMC5755158; doi:10.1186/s40168-017-0384-1)
Supplement: Supplementary file 2 — Vaccinations. Table S3. Injectable medications. Table S4. Water medications. (PDF 36 kb) [file 40168_2017_384_MOESM2_ESM.pdf]

Table S2. Vaccinations

Condition	Timing
Mycoplasma Hyopneumoniae	Processing (~4 days old)
Porcine Circovirus Type 2 (PCV2)	Weaning
Porcine Respiratory Syndrome (PRRS)	10-14 days post-weaning
Porcine Circovirus	14-17 days post PRRS vaccination
Mycoplasma Hyopneumoniae	
Ileitis	~6 weeks post-weaning
Erysipelas	

Table S3. Injectable medications

Condition	Product	Timing
Respiratory, Diarrhea, Lameness	Excede	Weaning to 8 weeks post-weaning
Respiratory	Biomycin 200	8-14 weeks post-weaning
Respiratory	Lincocin 300	14 weeks post-weaning to end of study
Diarrhea, Lameness	Lincocin 300	8 weeks post-weaning to end of study
Lameness	Dexamethasone	Weaning to 14 weeks post-weaning

Table S4. Water medications

Condition	Product	Timing
Diarrhea	Neomycin	Weaning
Respiratory, Diarrhea	Oxytetracycline (OTC)<br>Denagard	As Needed
Respiratory, Diarrhea, Lameness	Linco Soluble	As Needed
