# Supplementary material for: Host contributes to longitudinal diversity of fecal microbiota in swine selected for lean growth
Source: Microbiome. 2018 Jan 4;6:4. doi: 10.1186/s40168-017-0384-1 (PMC5755158; doi:10.1186/s40168-017-0384-1)
Supplement: Supplementary file 4 — Proportion of OTU counts at weaning, week 15, and off-test by phylum. Table S7. Proportion of OTU counts at weaning, week 15, and off-test by class. Table S8. Proportion of OTU counts at weaning, week 15, and off-test by order. Table S9. Proportion of OTU counts at weaning, week 15, and off-test by family. Table S10. Proportion of OTU counts at weaning, week 15, and off-test by genus. Table S11. Proportion of OTU counts at weaning, week 15, and off-test by species. (PDF 141 kb) [file 40168_2017_384_MOESM4_ESM.pdf]

Table S6. Proportion of OTU counts at weaning, week 15, and off-test by phylum

| Phylum | Wean | Week15 | Off-Test |
|---|---|---|---|
| Firmicutes | 4.54E+03 | 7.34E+03 | 7.49E+03 |
| Bacteroidetes | 2.82E+03 | 2.19E+03 | 1.81E+03 |
| Proteobacteria | 1.63E+03 | 1.38E+02 | 1.66E+02 |
| Fusobacteria | 3.30E+02 | 2.95E+00 | 1.40E+01 |
| Spirochaetes | 1.61E+02 | 6.81E+01 | 1.49E+02 |
| Actinobacteria | 9.69E+01 | 3.58E+01 | 9.58E+01 |
| Synergistetes | 1.07E+01 | 1.73E-01 | 4.79E-02 |
| Elusimicrobia | 8.44E+00 | 1.09E+00 | 8.18E-01 |
| Deferribacteres | 6.47E+00 | 7.88E-01 | 2.02E-01 |
| Chlamydiae | 4.69E+00 | 6.12E-01 | 1.96E-01 |
| Verrucomicrobia | 2.03E+00 | 7.72E-03 | 0.00E+00 |
| Lentisphaerae | 1.67E+00 | 1.98E-01 | 2.83E-01 |
| Fibrobacteres | 1.82E-01 | 3.83E+00 | 6.76E+00 |
| Tenericutes | 1.16E-02 | 6.49E-01 | 2.14E+00 |
| Unassigned | 3.86E+02 | 2.12E+02 | 2.62E+02 |

Table S7. Proportion of OTU counts at weaning, week 15, and off-test by class

| Class | Wean | Week15 | Off-Test |
|---|---|---|---|
| Clostridia | 3.40E+03 | 5.02E+03 | 5.52E+03 |
| Fusobacteriia | 3.30E+02 | 2.95E+00 | 1.40E+01 |
| Spirochaetia | 1.61E+02 | 6.81E+01 | 1.49E+02 |
| Bacteroidia | 2.47E+03 | 1.97E+03 | 1.36E+03 |
| Fibrobacteria | 1.82E-01 | 3.83E+00 | 6.76E+00 |
| Bacilli | 7.46E+02 | 1.75E+03 | 1.21E+03 |
| Lentisphaeria | 1.67E+00 | 1.98E-01 | 2.83E-01 |
| Gammaproteobacteria | 1.06E+03 | 6.40E+01 | 4.49E+01 |
| Negativicutes | 9.91E+01 | 2.97E+02 | 7.50E+01 |
| Erysipelotrichia | 4.00E+01 | 1.61E+02 | 4.66E+02 |
| Epsilonproteobacteria | 4.13E+02 | 3.17E+01 | 8.31E+01 |
| Actinobacteria_1760 | 9.69E+01 | 3.58E+01 | 9.58E+01 |
| Deltaproteobacteria | 1.18E+02 | 2.61E+01 | 2.60E+01 |
| Betaproteobacteria | 4.18E+01 | 9.94E+00 | 8.92E+00 |
| Elusimicrobia_641853 | 8.44E+00 | 1.09E+00 | 8.18E-01 |
| Synergistia | 1.07E+01 | 1.73E-01 | 4.79E-02 |
| Mollicutes | 1.16E-02 | 6.49E-01 | 2.14E+00 |
| Chlamydiia | 4.69E+00 | 6.12E-01 | 1.96E-01 |
| Verrucomicrobiae | 1.47E+00 | 0.00E+00 | 0.00E+00 |
| Deferribacteres_68337 | 6.47E+00 | 7.88E-01 | 2.02E-01 |
| Flavobacteriia | 7.69E-01 | 1.24E-02 | 7.86E-04 |
| Unassigned | 1.00E+03 | 5.56E+02 | 9.32E+02 |

Table S8. Proportion of OTU counts at weaning, week 15, and off-test by order

| Order | Wean | Week15 | Off-Test |
|---|---|---|---|
| Clostridiales | 3.39E+03 | 5.01E+03 | 5.49E+03 |
| Fusobacteriales | 3.30E+02 | 2.95E+00 | 1.40E+01 |
| Spirochaetales | 1.61E+02 | 6.81E+01 | 1.49E+02 |
| Bacteroidales | 2.47E+03 | 1.97E+03 | 1.36E+03 |
| Fibrobacterales | 1.82E-01 | 3.83E+00 | 6.76E+00 |
| Lactobacillales | 7.38E+02 | 1.74E+03 | 1.18E+03 |
| Victivallales | 1.67E+00 | 1.98E-01 | 2.83E-01 |
| Enterobacteriales | 7.80E+02 | 1.75E+01 | 2.30E+01 |
| Selenomonadales | 9.91E+01 | 2.97E+02 | 7.50E+01 |
| Erysipelotrichales | 4.00E+01 | 1.61E+02 | 4.66E+02 |
| Campylobacterales | 4.13E+02 | 3.17E+01 | 8.31E+01 |
| Actinomycetales | 4.35E+01 | 1.57E+01 | 6.74E+01 |
| Coriobacteriales | 4.03E+01 | 1.17E+01 | 1.37E+01 |
| Desulfovibrionales | 1.12E+02 | 1.90E+01 | 1.82E+01 |
| Pasteurellales | 2.37E+02 | 1.57E+00 | 2.73E+00 |
| Aeromonadales | 3.68E+01 | 4.44E+01 | 1.45E+01 |
| Bacillales | 8.03E+00 | 9.55E+00 | 3.56E+01 |
| Burkholderiales | 4.17E+01 | 9.31E+00 | 6.82E+00 |
| Elusimicrobiales | 8.44E+00 | 1.09E+00 | 8.18E-01 |
| Synergistales | 1.07E+01 | 1.73E-01 | 4.79E-02 |
| Bifidobacteriales | 1.27E+01 | 1.33E-01 | 3.10E-01 |
| Anaeroplasmatales | 1.16E-02 | 6.49E-01 | 2.14E+00 |
| Chlamydiales | 4.69E+00 | 6.12E-01 | 1.96E-01 |
| Pseudomonadales | 1.66E+00 | 4.79E-01 | 4.70E+00 |
| Verrucomicrobiales | 1.47E+00 | 0.00E+00 | 0.00E+00 |
| Deferribacterales | 6.47E+00 | 7.88E-01 | 2.02E-01 |
| Flavobacteriales | 7.69E-01 | 1.24E-02 | 7.86E-04 |
| Neisseriales | 2.99E-02 | 1.36E-01 | 8.59E-01 |
| Rhodocyclales | 8.05E-02 | 4.99E-01 | 1.24E+00 |
| Unassigned | 1.01E+03 | 5.85E+02 | 9.80E+02 |

Table S9. Proportion of OTU counts at weaning, week 15, and off-test by family

| Family | Wean | Week 15 | Off-Test |
|---|---|---|---|
| Prevotellaceae | 9.89E+02 | 1.46E+03 | 7.42E+02 |
| Clostridiaceae | 8.22E+02 | 1.56E+03 | 1.79E+03 |
| Enterobacteriaceae | 7.80E+02 | 1.75E+01 | 2.30E+01 |
| Bacteroidaceae | 7.30E+02 | 1.48E+01 | 4.01E+01 |
| Ruminococcaceae | 5.32E+02 | 4.63E+02 | 2.47E+02 |
| Porphyromonadaceae | 3.65E+02 | 2.94E+02 | 2.92E+02 |
| Fusobacteriaceae | 3.30E+02 | 2.95E+00 | 1.40E+01 |
| Campylobacteraceae | 2.97E+02 | 2.59E+01 | 7.75E+01 |
| Eubacteriaceae | 2.71E+02 | 1.92E+02 | 1.48E+02 |
| Lactobacillaceae | 2.68E+02 | 7.85E+02 | 2.68E+02 |
| Streptococcaceae | 2.57E+02 | 9.35E+02 | 8.77E+02 |
| Pasteurellaceae | 2.37E+02 | 1.57E+00 | 2.73E+00 |
| Enterococcaceae | 2.10E+02 | 7.32E-01 | 5.60E+00 |
| Spirochaetaceae | 1.61E+02 | 6.81E+01 | 1.49E+02 |
| Lachnospiraceae | 1.35E+02 | 3.43E+02 | 2.53E+02 |
| Helicobacteraceae | 1.16E+02 | 5.82E+00 | 5.56E+00 |
| Unclassified_clostridiales | 1.07E+02 | 7.45E+00 | 5.37E+00 |
| Desulfovibrionaceae | 1.05E+02 | 1.59E+01 | 1.45E+01 |
| Veillonellaceae | 6.64E+01 | 2.73E+02 | 5.48E+01 |
| Oscillospiraceae | 5.50E+01 | 3.77E+01 | 3.89E+01 |
| Clostridiales_family_xi_incertae_sedis | 4.92E+01 | 1.96E+01 | 9.75E+01 |
| Coriobacteriaceae | 4.03E+01 | 1.17E+01 | 1.37E+01 |
| Erysipelotrichaceae | 4.00E+01 | 1.61E+02 | 4.66E+02 |
| Rikenellaceae | 3.80E+01 | 8.38E-01 | 1.35E+00 |
| Succinivibrionaceae | 3.68E+01 | 4.44E+01 | 1.45E+01 |
| Acidaminococcaceae | 3.27E+01 | 2.37E+01 | 2.02E+01 |
| Actinomycetaceae | 3.01E+01 | 1.15E+01 | 3.92E+01 |
| Clostridiales_family_xiii_incertae_sedis | 2.35E+01 | 1.44E+01 | 8.78E+00 |
| Sutterellaceae | 2.22E+01 | 5.80E+00 | 3.72E+00 |
| Peptostreptococcaceae | 1.84E+01 | 4.52E+02 | 5.57E+02 |
| Peptococcaceae | 1.33E+01 | 9.44E+00 | 2.49E+01 |
| Bifidobacteriaceae | 1.27E+01 | 1.33E-01 | 3.10E-01 |
| Synergistaceae | 1.07E+01 | 1.73E-01 | 4.79E-02 |
| Elusimicrobiaceae | 8.44E+00 | 1.09E+00 | 8.18E-01 |
| Staphylococcaceae | 7.02E+00 | 2.56E+00 | 5.76E+00 |
| Deferribacteraceae | 6.47E+00 | 7.88E-01 | 2.02E-01 |
| Chlamydiaceae | 4.69E+00 | 6.12E-01 | 1.96E-01 |
| Aerococcaceae | 3.09E+00 | 9.31E+00 | 2.13E+01 |
| Comamonadaceae | 2.73E+00 | 1.54E-02 | 2.67E-02 |
| Corynebacteriaceae | 2.73E+00 | 3.08E+00 | 3.33E+00 |
| Micrococcaceae | 2.72E+00 | 1.74E-01 | 2.84E-01 |

| | | | |
|---|---|---|---|
| Victivallaceae | 1.67E+00 | 1.98E-01 | 2.83E-01 |
| Moraxellaceae | 1.66E+00 | 4.79E-01 | 4.70E+00 |
| Verrucomicrobiaceae | 1.47E+00 | 0.00E+00 | 0.00E+00 |
| Leuconostocaceae | 1.18E+00 | 1.07E+01 | 1.58E+00 |
| Oxalobacteraceae | 9.72E-01 | 1.20E+00 | 1.38E+00 |
| Bacillaceae | 9.65E-01 | 8.13E-01 | 2.36E-01 |
| Christensenellaceae | 9.00E-01 | 6.95E-03 | 3.93E-03 |
| Flavobacteriaceae | 7.69E-01 | 1.24E-02 | 7.86E-04 |
| Fibrobacteraceae | 1.82E-01 | 3.83E+00 | 6.76E+00 |
| Rhodocyclaceae | 8.05E-02 | 4.99E-01 | 1.24E+00 |
| Planococcaceae | 3.98E-02 | 6.07E+00 | 2.87E+01 |
| Neisseriaceae | 2.99E-02 | 1.36E-01 | 8.59E-01 |
| Anaeroplasmataceae | 1.16E-02 | 6.49E-01 | 2.14E+00 |
| Unassigned | 2.75E+03 | 2.70E+03 | 3.62E+03 |

Table S10. Proportion of OTU counts at weaning, week 15, and off-test by genus

| Genus | Wean | Week 15 | Off-Test |
|---|---|---|---|
| Clostridium | 8.18E+02 | 1.55E+03 | 1.78E+03 |
| Escherichia | 7.73E+02 | 1.74E+01 | 2.30E+01 |
| Bacteroides | 7.30E+02 | 1.48E+01 | 4.01E+01 |
| Prevotella | 6.78E+02 | 1.31E+03 | 6.74E+02 |
| Ruminococcus | 3.41E+02 | 2.43E+02 | 1.68E+02 |
| Fusobacterium | 3.24E+02 | 2.94E+00 | 1.40E+01 |
| Campylobacter | 2.97E+02 | 2.59E+01 | 7.75E+01 |
| Eubacterium | 2.71E+02 | 1.92E+02 | 1.48E+02 |
| Lactobacillus | 2.57E+02 | 7.84E+02 | 2.68E+02 |
| Streptococcus | 2.57E+02 | 9.35E+02 | 8.77E+02 |
| Enterococcus | 2.10E+02 | 7.32E-01 | 5.60E+00 |
| Actinobacillus | 1.60E+02 | 8.19E-01 | 2.70E+00 |
| Treponema | 1.52E+02 | 6.74E+01 | 1.48E+02 |
| Helicobacter | 1.16E+02 | 5.82E+00 | 5.56E+00 |
| Subdoligranulum | 1.04E+02 | 4.22E+01 | 1.21E+01 |
| Desulfovibrio | 9.37E+01 | 1.58E+01 | 1.45E+01 |
| Unclassified_pasteurellaceae | 7.22E+01 | 6.92E-01 | 2.59E-02 |
| Parabacteroides | 7.22E+01 | 1.16E+00 | 4.08E-01 |
| Oscillibacter | 5.50E+01 | 3.77E+01 | 3.89E+01 |
| Butyricimonas | 5.11E+01 | 4.39E-01 | 2.75E-02 |
| Alloprevotella | 4.89E+01 | 1.86E+01 | 1.88E+01 |
| Roseburia | 4.64E+01 | 3.22E+01 | 2.13E+01 |
| Alistipes | 3.80E+01 | 8.38E-01 | 1.35E+00 |
| Anaerococcus | 3.43E+01 | 1.45E+01 | 8.01E+01 |
| Faecalibacterium | 3.31E+01 | 1.01E+02 | 3.03E+01 |

| | | | |
|---|---|---|---|
| Blautia | 3.04E+01 | 4.46E+01 | 3.67E+01 |
| Dorea | 2.83E+01 | 1.19E+01 | 7.47E+00 |
| Unclassified_erysipelotrichaceae | 2.70E+01 | 6.10E+00 | 2.54E+00 |
| Phascolarctobacterium | 2.63E+01 | 2.16E+01 | 2.01E+01 |
| Megasphaera | 2.41E+01 | 8.80E+01 | 3.77E+00 |
| Mogibacterium | 2.35E+01 | 1.44E+01 | 8.78E+00 |
| Sutterella | 2.16E+01 | 5.53E+00 | 3.57E+00 |
| Actinomyces | 2.09E+01 | 6.18E-02 | 1.48E+00 |
| Odoribacter | 2.08E+01 | 2.14E-01 | 5.03E-02 |
| Succinivibrio | 1.83E+01 | 4.38E+01 | 1.44E+01 |
| Anaerobiospirillum | 1.74E+01 | 6.23E-01 | 8.33E-02 |
| Olsenella | 1.63E+01 | 8.72E-01 | 1.41E-01 |
| Peptococcus | 1.33E+01 | 9.44E+00 | 2.49E+01 |
| Bifidobacterium | 1.27E+01 | 1.33E-01 | 3.10E-01 |
| Unclassified_peptostreptococcaceae | 1.17E+01 | 4.52E+02 | 5.57E+02 |
| Sharpea | 1.05E+01 | 3.55E-02 | 4.63E-02 |
| Coprococcus | 1.03E+01 | 8.99E+01 | 5.50E+01 |
| Bilophila | 1.02E+01 | 8.19E-02 | 7.86E-04 |
| Trueperella | 9.11E+00 | 7.41E-02 | 9.51E-02 |
| Elusimicrobium | 8.44E+00 | 1.09E+00 | 8.18E-01 |
| Dialister | 8.42E+00 | 5.21E+01 | 1.39E+00 |
| Anaerovibrio | 8.07E+00 | 3.22E+01 | 2.11E+01 |
| Collinsella | 7.89E+00 | 2.37E+00 | 8.78E-01 |
| Finegoldia | 7.76E+00 | 1.92E+00 | 2.48E+00 |
| Staphylococcus | 6.99E+00 | 2.37E+00 | 2.94E+00 |
| Synergistes | 6.71E+00 | 2.39E-02 | 2.91E-02 |
| Peptostreptococcus | 6.67E+00 | 2.55E-02 | 1.10E-02 |
| Mucispirillum | 6.47E+00 | 7.88E-01 | 2.02E-01 |
| Mitsuokella | 6.41E+00 | 3.37E+01 | 1.49E+01 |
| Turicibacter | 6.34E+00 | 1.50E+02 | 4.60E+02 |
| Veillonella | 5.09E+00 | 1.16E-02 | 2.08E-01 |
| Allisonella | 4.86E+00 | 2.49E+00 | 2.78E-01 |
| Chlamydia | 4.69E+00 | 6.12E-01 | 1.96E-01 |
| Acidaminococcus | 4.62E+00 | 2.14E+00 | 5.73E-02 |
| Peptoniphilus | 3.67E+00 | 2.99E+00 | 1.01E+01 |
| Pyramidobacter | 3.32E+00 | 1.48E-01 | 1.34E-02 |
| Howardella | 3.28E+00 | 1.27E-01 | 3.38E-02 |
| Comamonas | 2.73E+00 | 1.54E-02 | 2.67E-02 |
| Corynebacterium | 2.73E+00 | 3.08E+00 | 3.33E+00 |
| Rothia_32207 | 2.72E+00 | 1.74E-01 | 2.84E-01 |
| Aerococcus | 2.15E+00 | 9.09E+00 | 1.97E+01 |
| Anaerotruncus | 2.09E+00 | 6.95E-03 | 0.00E+00 |
| Parvimonas | 1.97E+00 | 1.37E-01 | 4.08E+00 |
| Porphyromonas | 1.94E+00 | 1.94E+00 | 3.32E+01 |

| | | | |
|---|---|---|---|
| Pseudoflavonifractor | 1.72E+00 | 3.16E-01 | 2.41E-01 |
| Victivallis | 1.67E+00 | 1.98E-01 | 2.83E-01 |
| Moraxella_475 | 1.48E+00 | 1.06E-01 | 2.91E-01 |
| Akkermansia | 1.47E+00 | 0.00E+00 | 0.00E+00 |
| Solobacterium | 1.40E+00 | 2.63E-02 | 5.82E-01 |
| Helcococcus | 1.32E+00 | 7.72E-03 | 1.66E-01 |
| Weissella | 1.18E+00 | 1.07E+01 | 1.58E+00 |
| Catenibacterium | 1.17E+00 | 2.07E+00 | 2.52E-01 |
| Ruminobacter | 1.03E+00 | 0.00E+00 | 0.00E+00 |
| Oxalobacter | 9.72E-01 | 1.20E+00 | 1.38E+00 |
| Bacillus_1386 | 9.65E-01 | 8.13E-01 | 2.36E-01 |
| Christensenella | 9.00E-01 | 6.95E-03 | 3.93E-03 |
| Atopobium | 7.93E-01 | 9.27E-03 | 5.00E-01 |
| Parasutterella | 6.38E-01 | 2.72E-01 | 1.48E-01 |
| Pediococcus | 3.30E-01 | 2.87E-01 | 4.34E-01 |
| Sporobacter | 3.20E-01 | 9.15E-01 | 9.25E-01 |
| Slackia | 1.83E-01 | 3.44E-01 | 3.93E-02 |
| Fibrobacter | 1.82E-01 | 3.83E+00 | 6.76E+00 |
| Acinetobacter | 1.78E-01 | 3.73E-01 | 4.41E+00 |
| Gallicola | 1.39E-01 | 1.24E-02 | 6.23E-01 |
| Anaerofustis | 1.23E-01 | 1.23E-01 | 1.44E-01 |
| Mobiluncus | 7.80E-02 | 9.83E+00 | 3.20E+01 |
| Lachnospira | 5.39E-02 | 2.55E+01 | 1.53E+01 |
| Flavonifractor | 5.31E-02 | 2.72E-01 | 6.83E-01 |
| Kurthia | 3.98E-02 | 5.77E+00 | 2.74E+01 |
| Facklamia | 2.49E-02 | 1.14E-01 | 1.44E+00 |
| Jeotgalicoccus | 2.41E-02 | 1.85E-01 | 1.32E+00 |
| Sporobacterium | 2.07E-02 | 7.04E-01 | 2.10E+01 |
| Cellulosilyticum | 1.99E-02 | 0.00E+00 | 5.84E-01 |
| Anaeroplasma | 1.16E-02 | 6.49E-01 | 2.14E+00 |
| Butyrivibrio | 1.16E-02 | 1.81E+00 | 6.21E-01 |
| Selenomonas | 1.08E-02 | 1.58E+00 | 2.60E-01 |
| Nosocomiicoccus | 7.47E-03 | 3.86E-03 | 1.50E+00 |
| Barnesiella | 5.81E-03 | 5.71E-01 | 3.29E+00 |
| Adlercreutzia | 4.98E-03 | 1.21E-01 | 3.89E-01 |
| Schwartzia_55506 | 1.66E-03 | 9.71E-01 | 5.03E-02 |
| Anaerostipes | 0.00E+00 | 2.00E+00 | 4.05E+00 |
| Unassigned | 3.47E+03 | 3.43E+03 | 4.11E+03 |

Table S11. Proportion of OTU counts at weaning, week 15, and off-test by species

| Species | Wean | Week 15 | Off-Test |
|---|---|---|---|
| Escherichia_coli | 7.66e+02 | 1.73e+01 | 2.27e+01 |
| Prevotella_sp_djf_ls16 | 3.92e+02 | 4.33e+01 | 1.27e+01 |
| Bacteroides_fragilis | 2.70e+02 | 2.14e-01 | 6.29e+00 |
| Campylobacter_jejuni | 1.70e+02 | 1.09e+00 | 1.41e+00 |
| Streptococcus_gallolyticus | 1.67e+02 | 9.19e+02 | 8.64e+02 |
| Eubacterium_coprostanoligenes | 1.59e+02 | 4.70e+01 | 5.39e+01 |
| Clostridium_disporicum | 1.46e+02 | 1.69e+02 | 2.52e+02 |
| Bacteroides_vulgatus | 1.37e+02 | 1.17e+00 | 9.40e-01 |
| Treponema_brennaborense | 1.31e+02 | 2.77e+00 | 7.97e-01 |
| Prevotella_sp_djf_b116 | 1.06e+02 | 1.75e+01 | 2.63e+00 |
| Enterococcus_faecalis | 1.05e+02 | 1.64e-01 | 1.78e-01 |
| Subdoligranulum_variabile | 1.04e+02 | 4.22e+01 | 1.21e+01 |
| Clostridium_lactatifermentans | 1.02e+02 | 3.08e+00 | 1.32e+00 |
| Enterococcus_cecorum | 1.01e+02 | 4.84e-01 | 5.07e+00 |
| Lactobacillus_reuteri | 1.00e+02 | 3.19e+02 | 1.20e+02 |
| Fusobacterium_perfoetens | 9.74e+01 | 2.40e-01 | 1.73e-01 |
| Actinobacillus_porcinus | 7.71e+01 | 4.09e-01 | 1.85e+00 |
| Actinobacillus_porcitonsillarum | 7.68e+01 | 3.68e-01 | 8.51e-01 |
| Unclassified_pasteurellaceae_aerogenes | 7.15e+01 | 6.90e-01 | 2.12e-02 |
| Streptococcus_suis | 6.56e+01 | 8.25e-01 | 2.56e+00 |
| Clostridium_scindens | 6.53e+01 | 2.91e-01 | 1.57e-03 |
| Alloprevotella_rava | 4.89e+01 | 1.86e+01 | 1.88e+01 |
| Eubacterium_sp_wal_14571 | 4.65e+01 | 6.39e+00 | 9.30e+00 |
| Butyricimonas_virosa | 4.61e+01 | 3.85e-01 | 1.73e-02 |
| Bacteroides_pyogenes | 4.53e+01 | 8.88e-02 | 3.93e-03 |
| Clostridium_bolteae | 4.40e+01 | 4.22e-01 | 1.02e-02 |
| Helicobacter_canadensis | 4.32e+01 | 7.04e-01 | 2.45e+00 |
| Lactobacillus_amylovorus | 4.29e+01 | 2.14e+02 | 1.17e+01 |
| Parabacteroides_distasonis | 3.94e+01 | 9.11e-02 | 2.36e-03 |
| Prevotella_copri | 3.93e+01 | 4.32e+02 | 8.41e+01 |
| Alistipes_shahii | 3.42e+01 | 3.41e-01 | 1.26e-02 |
| Lactobacillus_delbrueckii | 3.03e+01 | 1.56e+00 | 1.57e-01 |
| Dorea_longicatena | 2.75e+01 | 5.45e+00 | 7.33e+00 |
| Faecalibacterium_prausnitzii | 2.73e+01 | 9.66e+01 | 2.59e+01 |
| Phascolarctobacterium_succinatutens | 2.63e+01 | 2.16e+01 | 2.01e+01 |
| Eubacterium_desmolans | 2.61e+01 | 7.47e+01 | 4.65e+01 |
| Megasphaera_elsdenii | 2.40e+01 | 8.76e+01 | 3.75e+00 |
| Mogibacterium_neglectum | 2.35e+01 | 1.44e+01 | 8.78e+00 |
| Lactobacillus_gasseri | 2.24e+01 | 2.35e+02 | 1.32e+02 |
| Oscillibacter_sp_g2 | 2.24e+01 | 1.57e+00 | 8.64e-02 |
| Succinivibrio_dextrinosolvens | 1.83e+01 | 4.38e+01 | 1.44e+01 |

| | | | |
|---|---|---|---|
| Anaerococcus_sp_bg1 | 1.79e+01 | 3.41e-01 | 3.94e+00 |
| Anaerobiospirillum_succiniciproducens | 1.74e+01 | 6.23e-01 | 8.33e-02 |
| Odoribacter_splanchnicus | 1.72e+01 | 2.14e-01 | 4.95e-02 |
| Fusobacterium_necrophorum | 1.53e+01 | 2.00e-01 | 1.67e+00 |
| Sutterella_stercoricanis | 1.51e+01 | 5.47e+00 | 3.53e+00 |
| Clostridium_perfringens | 1.48e+01 | 1.61e-01 | 2.51e-02 |
| Bacteroides_heparinolyticus | 1.40e+01 | 3.86e-03 | 1.26e-02 |
| Peptococcus_niger | 1.33e+01 | 9.44e+00 | 2.49e+01 |
| Prevotella_sp_rs2 | 1.33e+01 | 2.19e+01 | 4.56e+00 |
| Bacteroides_coprophilus | 1.32e+01 | 5.48e-02 | 1.78e-01 |
| Bacteroides_uniformis | 1.24e+01 | 4.25e-02 | 1.26e-02 |
| Parabacteroides_goldsteinii | 1.23e+01 | 4.63e-03 | 0.00e+00 |
| Clostridium_hathewayi | 1.18e+01 | 6.62e+00 | 7.63e+00 |
| Fusobacterium_varium | 1.15e+01 | 1.07e-01 | 3.53e-01 |
| Unclassified_erysipelotrichaceae_biforme | 1.15e+01 | 6.09e+00 | 2.10e+00 |
| Oscillibacter_valericigenes | 1.13e+01 | 7.72e+00 | 1.44e+01 |
| Blautia_gnavus | 1.09e+01 | 1.16e-02 | 1.34e-02 |
| Sharpea_azabuensis | 1.05e+01 | 3.55e-02 | 4.63e-02 |
| Parabacteroides_merdae | 1.03e+01 | 1.24e-02 | 6.28e-03 |
| Bilophila_wadsworthia | 1.02e+01 | 8.19e-02 | 7.86e-04 |
| Anaerococcus_tetradius | 1.01e+01 | 2.37e+00 | 7.51e+00 |
| Coprococcus_comes | 9.57e+00 | 3.44e+01 | 2.79e+01 |
| Trueperella_pyogenes | 9.11e+00 | 7.41e-02 | 9.51e-02 |
| Clostridium_sp_id11 | 8.86e+00 | 1.87e+01 | 1.95e+01 |
| Unclassified_peptostreptococcaceae_glycolicum | 8.79e+00 | 4.07e+02 | 5.46e+02 |
| Elusimicrobium_minutum | 8.44e+00 | 1.09e+00 | 8.18e-01 |
| Lactobacillus_mucosae | 8.42e+00 | 1.28e+00 | 2.21e-01 |
| Unclassified_erysipelotrichaceae_ramosum | 8.35e+00 | 1.54e-03 | 2.72e-01 |
| Bacteroides_massiliensis | 8.21e+00 | 7.72e-04 | 7.86e-04 |
| Anaerovibrio_lipolyticus | 8.07e+00 | 3.22e+01 | 2.11e+01 |
| Bacteroides_plebeius | 7.92e+00 | 1.82e-01 | 3.37e+00 |
| Bacteroides_thetaiotaomicron | 7.83e+00 | 8.49e-03 | 3.93e-03 |
| Finegoldia_magna | 7.76e+00 | 1.92e+00 | 2.48e+00 |
| Lactobacillus_salivarius | 7.44e+00 | 3.60e-01 | 9.58e-02 |
| Collinsella_aerofaciens | 7.28e+00 | 2.37e+00 | 8.77e-01 |
| Unclassified_erysipelotrichaceae_pleomorphus | 7.16e+00 | 9.27e-03 | 1.66e-01 |
| Staphylococcus_saprophyticus | 6.98e+00 | 1.34e+00 | 2.18e+00 |
| Lactobacillus_murinus | 6.49e+00 | 1.24e-02 | 4.71e-03 |
| Sutterella_parvirubra | 6.48e+00 | 5.71e-02 | 3.93e-02 |
| Mucispirillum_schaedleri | 6.47e+00 | 7.88e-01 | 2.02e-01 |
| Turicibacter_sanguinis | 6.34e+00 | 1.50e+02 | 4.60e+02 |
| Helicobacter_rappini | 6.03e+00 | 8.26e-02 | 2.52e-01 |
| Treponema_porcinum | 6.02e+00 | 2.99e+01 | 4.44e+01 |
| Olsenella_umbonata | 5.97e+00 | 6.95e-03 | 1.10e-02 |

| | | | |
|---|---|---|---|
| Actinobacillus_pleuropneumoniae | 5.92e+00 | 4.17e-02 | 7.86e-04 |
| Eubacterium_hallii | 5.90e+00 | 3.34e+01 | 5.51e+00 |
| Clostridium_symbiosum | 5.30e+00 | 4.05e+00 | 3.70e+00 |
| Blautia_obeum | 5.15e+00 | 1.39e+01 | 3.04e+00 |
| Blautia_glucerasea | 5.02e+00 | 1.54e-03 | 0.00e+00 |
| Allisonella_histaminiformans | 4.86e+00 | 2.49e+00 | 2.78e-01 |
| Chlamydia_suis | 4.69e+00 | 6.12e-01 | 1.96e-01 |
| Roseburia_intestinalis | 4.68e+00 | 2.17e+01 | 1.82e+01 |
| Bacteroides_ovatus | 4.51e+00 | 6.95e-03 | 3.93e-03 |
| Mitsuokella_jalaludinii | 4.13e+00 | 3.37e+01 | 1.48e+01 |
| Clostridium_butyricum | 4.05e+00 | 1.01e+02 | 1.15e+02 |
| Blautia_torques | 3.88e+00 | 3.78e+00 | 1.88e-01 |
| Clostridium_hylemonae | 3.85e+00 | 1.12e+00 | 3.94e+00 |
| Helicobacter_bilis | 3.33e+00 | 2.32e-03 | 5.40e-01 |
| Pyramidobacter_piscolens | 3.32e+00 | 1.48e-01 | 1.34e-02 |
| Howardella_ureilytica | 3.28e+00 | 1.27e-01 | 3.38e-02 |
| Eubacterium_limosum | 3.17e+00 | 2.16e-02 | 4.71e-03 |
| Eubacterium_pyruvativorans | 3.10e+00 | 2.30e-01 | 6.76e-02 |
| Bacteroides_caccae | 3.07e+00 | 1.08e-02 | 0.00e+00 |
| Ruminococcus_bromii | 2.90e+00 | 1.18e+01 | 1.58e+01 |
| Anaerococcus_hydrogenalis | 2.87e+00 | 1.34e-01 | 9.51e-02 |
| Lactobacillus_coleohominis | 2.77e+00 | 1.61e-01 | 2.36e-03 |
| Comamonas_aquatica | 2.73e+00 | 1.54e-02 | 2.67e-02 |
| Streptococcus_porci | 2.70e+00 | 3.09e-02 | 1.41e+00 |
| Mitsuokella_multacida | 2.28e+00 | 2.55e-02 | 1.65e-02 |
| Ruminococcus_sp_ce2 | 2.23e+00 | 8.86e+01 | 1.90e+01 |
| Clostridium_lavalense | 2.13e+00 | 1.44e-01 | 1.67e+00 |
| Rothia_32207_nasimurium | 2.02e+00 | 1.44e-01 | 2.06e-01 |
| Lactobacillus_agilis | 2.01e+00 | 1.53e-01 | 6.17e-01 |
| Fusobacterium_mortiferum | 1.97e+00 | 2.63e-02 | 1.04e-01 |
| Parvimonas_micra | 1.97e+00 | 1.37e-01 | 4.08e+00 |
| Pseudoflavonifractor_capillosus | 1.72e+00 | 3.16e-01 | 2.41e-01 |
| Victivallis_vadensis | 1.67e+00 | 1.98e-01 | 2.83e-01 |
| Clostridium_sp_nml_04a032 | 1.58e+00 | 3.30e-01 | 4.35e-01 |
| Corynebacterium_glucuronolyticum | 1.50e+00 | 1.13e+00 | 8.02e-01 |
| Akkermansia_muciniphila | 1.47e+00 | 0.00e+00 | 0.00e+00 |
| Solobacterium_moorei | 1.40e+00 | 2.63e-02 | 5.82e-01 |
| Treponema_bryantii | 1.24e+00 | 2.60e+00 | 9.57e+00 |
| Ruminococcus_sp_ye281 | 1.22e+00 | 1.09e+00 | 3.62e+00 |
| Ruminococcus_flavefaciens | 1.21e+00 | 5.29e+00 | 1.38e+01 |
| Anaerotruncus_sp_nml_070203 | 1.20e+00 | 6.18e-03 | 0.00e+00 |
| Ruminococcus_callidus | 1.18e+00 | 4.91e+00 | 1.21e+01 |
| Catenibacterium_mitsuokai | 1.17e+00 | 2.07e+00 | 2.52e-01 |
| Synergistes_sp_nml96a088 | 1.15e+00 | 3.09e-03 | 3.93e-03 |

| | | | |
|---|---|---|---|
| Clostridium_septicum | 1.12e+00 | 2.01e-02 | 1.71e-01 |
| Bacteroides_stercoris | 1.04e+00 | 4.63e-03 | 4.71e-03 |
| Ruminobacter_amylophilus | 1.03e+00 | 0.00e+00 | 0.00e+00 |
| Oxalobacter_formigenes | 9.72e-01 | 1.20e+00 | 1.38e+00 |
| Bacillus_1386_amyloliquefaciens | 9.65e-01 | 8.49e-03 | 1.57e-02 |
| Christensenella_minuta | 9.00e-01 | 6.95e-03 | 3.93e-03 |
| Anaerotruncus_colihominis | 8.88e-01 | 7.72e-04 | 0.00e+00 |
| Eubacterium_siraeum | 8.85e-01 | 4.79e+00 | 5.96e+00 |
| Helcococcus_sueciensis | 8.58e-01 | 7.72e-04 | 6.28e-03 |
| Veillonella_sp_oral_taxon_780 | 8.27e-01 | 1.54e-03 | 1.10e-02 |
| Blautia_producta | 8.26e-01 | 5.80e-01 | 2.75e+00 |
| Atopobium_minutum | 7.93e-01 | 9.27e-03 | 5.00e-01 |
| Dorea_formicigenerans | 7.78e-01 | 6.41e+00 | 1.39e-01 |
| Alistipes_putredinis | 7.59e-01 | 0.00e+00 | 0.00e+00 |
| Unclassified_pasteurellaceae_mairii | 7.36e-01 | 1.54e-03 | 4.71e-03 |
| Coprococcus_catus | 6.80e-01 | 5.01e+01 | 2.44e+01 |
| Aerococcus_viridans | 6.79e-01 | 2.92e+00 | 8.45e+00 |
| Faecalibacterium_sp_djf_vr20 | 6.68e-01 | 7.87e-01 | 1.21e+00 |
| Clostridium_sp_id5 | 6.58e-01 | 7.10e-02 | 7.49e-01 |
| Parasutterella_secunda | 6.38e-01 | 2.72e-01 | 1.48e-01 |
| Collinsella_stercoris | 6.10e-01 | 0.00e+00 | 7.86e-04 |
| Unclassified_peptostreptococcaceae_hiranonis | 5.74e-01 | 0.00e+00 | 0.00e+00 |
| Weissella_paramesenteroides | 5.42e-01 | 8.59e+00 | 3.21e-01 |
| Treponema_berlinense | 5.34e-01 | 2.32e+00 | 7.45e-01 |
| Bacteroides_nordii | 5.33e-01 | 3.09e-03 | 0.00e+00 |
| Bacteroides_eggerthii | 5.16e-01 | 6.18e-03 | 0.00e+00 |
| Treponema_succinifaciens | 5.01e-01 | 2.01e+00 | 7.33e-01 |
| Prevotella_buccalis | 3.34e-01 | 3.60e-01 | 1.24e+01 |
| Pediococcus_pentosaceus | 3.30e-01 | 2.87e-01 | 4.34e-01 |
| Sporobacter_termitidis | 3.20e-01 | 9.15e-01 | 9.25e-01 |
| Campylobacter_cuniculorum | 2.91e-01 | 5.79e-02 | 2.53e-01 |
| Clostridium_nexile | 2.61e-01 | 2.55e-02 | 2.13e-01 |
| Ruminococcus_sp_djf_vr67 | 1.97e-01 | 1.51e+00 | 8.70e-01 |
| Clostridium_beijerinckii | 1.88e-01 | 2.89e-01 | 1.78e-01 |
| Slackia_isoflavoniconvertens | 1.83e-01 | 3.44e-01 | 3.93e-02 |
| Porphyromonas_somerae | 1.74e-01 | 6.27e-01 | 7.33e+00 |
| Fibrobacter_intestinalis | 1.69e-01 | 3.06e+00 | 2.97e+00 |
| Gallicola_barnesae | 1.39e-01 | 1.24e-02 | 6.23e-01 |
| Acinetobacter_lwoffii | 1.36e-01 | 1.24e-01 | 2.98e+00 |
| Ruminococcus_sp_nml_000124 | 1.31e-01 | 2.63e+00 | 2.28e+00 |
| Anaerofustis_stercorihominis | 1.23e-01 | 1.23e-01 | 1.44e-01 |
| Campylobacter_hominis | 7.88e-02 | 5.75e+00 | 3.14e+01 |
| Clostridium_sp_yit_12070 | 7.88e-02 | 2.53e-01 | 1.81e-01 |
| Eubacterium_sp_cl1013 | 7.14e-02 | 1.94e+00 | 4.03e-01 |

| | | | |
|---|---|---|---|
| Treponema_sp_cc2 | 7.14e-02 | 3.07e-01 | 3.27e+00 |
| Anaerococcus_vaginalis | 7.05e-02 | 1.38e+00 | 4.68e+00 |
| Clostridium_clostridioforme | 6.89e-02 | 6.61e+00 | 1.04e+00 |
| Clostridium_sp_shc10 | 5.81e-02 | 4.83e+00 | 3.38e+00 |
| Lachnospira_pectinoschiza | 5.39e-02 | 2.55e+01 | 1.53e+01 |
| Flavonifractor_plautii | 5.31e-02 | 2.72e-01 | 6.83e-01 |
| Eubacterium_ventriosum | 4.40e-02 | 3.07e-01 | 1.96e-01 |
| Kurthia_gibsonii | 3.98e-02 | 5.77e+00 | 2.74e+01 |
| Eubacterium_sp_oral_strain_a35mt | 3.90e-02 | 2.24e-02 | 1.15e+00 |
| Ruminococcus_sp_zs215 | 2.74e-02 | 9.67e+00 | 5.74e+00 |
| Facklamia_tabacinasalis | 2.49e-02 | 1.14e-01 | 1.44e+00 |
| Corynebacterium_glutamicum | 2.24e-02 | 2.08e-01 | 2.71e-01 |
| Sporobacterium_sp_wal_1855d | 2.07e-02 | 7.04e-01 | 2.10e+01 |
| Cellulosilyticum_ruminicola | 1.99e-02 | 0.00e+00 | 5.84e-01 |
| Coprococcus_eutactus | 1.49e-02 | 5.40e+00 | 2.71e+00 |
| Treponema_saccharophilum | 1.41e-02 | 8.42e-02 | 5.85e-01 |
| Fibrobacter_succinogenes | 1.24e-02 | 7.63e-01 | 3.79e+00 |
| Butyrivibrio_crossotus | 1.16e-02 | 1.81e+00 | 6.21e-01 |
| Selenomonas_ruminantium | 1.08e-02 | 1.58e+00 | 2.60e-01 |
| Clostridium_sp_l250 | 7.47e-03 | 4.06e-01 | 4.40e-02 |
| Corynebacterium_stationis | 7.47e-03 | 5.88e-01 | 3.68e-01 |
| Nosocomiicoccus_ampullae | 7.47e-03 | 3.86e-03 | 1.50e+00 |
| Adlercreutzia_equolifaciens | 4.98e-03 | 1.21e-01 | 3.89e-01 |
| Eubacterium_rectale | 2.49e-03 | 1.18e+00 | 1.96e-01 |
| Schwartzia_55506_succinivorans | 1.66e-03 | 9.71e-01 | 5.03e-02 |
| Bacteroides_galacturonicus | 0.00e+00 | 1.12e+00 | 2.95e-01 |
| Clostridium_methylpentosum | 0.00e+00 | 2.37e-01 | 2.59e-01 |
| Porphyromonas_bennonis | 0.00e+00 | 7.80e-02 | 7.97e-01 |
| Ruminococcus_albus | 0.00e+00 | 5.71e-01 | 8.99e-01 |
| Unassigned | 5.31e+03 | 5.91e+03 | 6.59e+03 |
