# Supplementary material for: Host contributes to longitudinal diversity of fecal microbiota in swine selected for lean growth
Source: Microbiome. 2018 Jan 4;6:4. doi: 10.1186/s40168-017-0384-1 (PMC5755158; doi:10.1186/s40168-017-0384-1)
Supplement: Supplementary file 5 — P values from Kruskal-Wallis tests for differences in abundance between pairs of time points at the genus level. (PDF 71 kb) [file 40168_2017_384_MOESM5_ESM.pdf]

Table S12. P-values from Kruskal-Wallis tests for differences in abundance between pairs of time points at the genus level.

Genus	Weaning vs Wk15	Weaning vs Off-test	Wk15 vs Off-test
Acidaminococcus	1.08E-38	5.48E-67	1.08E-180
Acinetobacter	8.57E-01	3.34E-27	1.18E-28
Actinobacillus	0.00E+00	1.29E-254	1.38E-24
Actinomyces	3.25E-179	7.14E-87	1.21E-35
Adlercreutzia	2.01E-27	1.26E-91	3.93E-36
Aerococcus	5.25E-108	2.68E-202	1.43E-28
Akkermansia	7.70E-23	1.74E-22	NaN
Alistipes	2.51E-298	1.10E-216	7.08E-57
Allisonella	1.13E-27	7.15E-37	9.22E-151
Alloprevotella	2.10E-03	5.55E-14	2.29E-14
Anaerobiospirillum	4.32E-47	1.65E-91	5.23E-19
Anaerococcus	5.99E-10	9.30E-42	9.41E-98
Anaerofustis	8.05E-01	8.86E-02	1.37E-01
Anaeroplasma	6.15E-90	2.67E-238	2.80E-79
Anaerostipes	6.51E-164	1.51E-295	7.98E-54
Anaerotruncus	7.29E-129	2.02E-128	1.61E-01
Anaerovibrio	5.98E-268	5.60E-228	2.11E-24
Atopobium	2.02E-50	1.92E-22	3.42E-11
Bacillus_1386	6.74E-03	5.97E-05	9.52E-12
Bacteroides	0.00E+00	0.00E+00	1.69E-139
Barnesiella	1.88E-52	1.82E-287	2.22E-180
Bifidobacterium	6.52E-181	4.52E-126	1.30E-16
Bilophila	0.00E+00	0.00E+00	2.07E-02
Blautia	3.85E-221	1.59E-184	3.64E-42
Butyricimonas	0.00E+00	0.00E+00	1.02E-02
Butyrivibrio	2.81E-203	3.86E-83	1.13E-52
Campylobacter	6.65E-117	4.99E-27	1.75E-77
Catenibacterium	5.22E-44	7.10E-22	1.80E-104
Cellulosilyticum	5.76E-06	5.68E-74	1.12E-91
Chlamydia	1.50E-11	1.14E-01	4.79E-20
Christensenella	3.62E-117	2.17E-117	4.21E-01
Clostridium	4.30E-171	1.07E-253	5.88E-28
Collinsella	1.80E-42	9.20E-151	8.77E-78
Comamonas	3.09E-11	1.21E-05	5.13E-03
Coprococcus	0.00E+00	0.00E+00	1.15E-135
Corynebacterium	7.46E-02	3.91E-11	1.59E-06
Desulfovibrio	8.73E-291	2.38E-297	9.52E-01
Dialister	2.27E-229	3.18E-40	0.00E+00
Dorea	1.81E-20	2.97E-46	1.65E-21

Elusimicrobium	2.07E-10	5.48E-15	2.20E-02
Enterococcus	2.39E-267	1.95E-175	1.30E-21
Escherichia	0.00E+00	0.00E+00	3.31E-39
Eubacterium	1.68E-02	2.76E-21	3.29E-86
Facklamia	2.59E-07	3.17E-104	1.53E-80
Faecalibacterium	6.57E-198	1.25E-26	1.15E-218
Fibrobacter	5.88E-277	1.71E-269	1.60E-08
Finegoldia	3.12E-28	3.92E-13	3.56E-05
Flavonifractor	5.84E-35	2.44E-126	6.28E-45
Fusobacterium	0.00E+00	9.19E-276	2.00E-29
Gallicola	5.81E-03	7.38E-28	1.37E-39
Helcococcus	8.79E-49	3.34E-23	2.35E-11
Helicobacter	2.66E-114	3.10E-160	4.96E-24
Howardella	2.00E-234	5.54E-274	3.44E-13
Jeotgalicoccus	1.03E-13	3.00E-57	3.10E-25
Kurthia	9.22E-96	6.48E-64	1.60E-06
Lachnospira	0.00E+00	0.00E+00	4.22E-46
Lactobacillus	3.49E-153	1.49E-09	2.19E-125
Megasphaera	3.72E-200	3.83E-153	0.00E+00
Mitsuokella	0.00E+00	1.10E-261	3.87E-131
Mobiluncus	1.30E-253	0.00E+00	2.62E-92
Mogibacterium	7.05E-12	9.19E-55	3.16E-67
Moraxella_475	2.45E-61	6.43E-22	2.48E-15
Mucispirillum	3.85E-01	7.39E-25	1.29E-27
Nosocomiicoccus	9.07E-01	1.20E-120	4.40E-129
Odoribacter	7.12E-231	5.01E-224	1.67E-01
Olsenella	2.12E-01	2.67E-66	6.07E-98
Oscillibacter	6.27E-06	1.74E-04	4.23E-04
Oxalobacter	8.77E-05	3.91E-03	5.55E-01
Parabacteroides	8.82E-227	1.49E-276	1.50E-13
Parasutterella	6.40E-04	3.65E-02	4.69E-09
Parvimonas	2.65E-22	5.18E-02	2.28E-30
Pediococcus	8.33E-02	7.85E-03	3.30E-01
Peptococcus	9.46E-05	2.43E-121	4.74E-215
Peptoniphilus	1.66E-03	1.88E-72	5.00E-63
Peptostreptococcus	1.60E-63	7.45E-70	3.54E-02
Phascolarctobacterium	4.05E-09	1.68E-15	3.57E-03
Porphyromonas	1.09E-08	5.51E-204	1.77E-177
Prevotella	1.36E-133	1.53E-06	7.71E-197
Pseudoflavonifractor	1.86E-13	3.20E-23	1.43E-03
Pyramidobacter	1.22E-87	3.52E-82	6.56E-02
Roseburia	8.46E-43	1.14E-12	2.19E-37
Rothia_32207	1.77E-195	3.99E-158	1.16E-06

Ruminobacter	3.36E-07	4.22E-07	NaN
Ruminococcus	1.99E-03	1.39E-06	1.91E-113
Schwartzia_55506	8.96E-83	4.82E-10	2.26E-62
Selenomonas	2.96E-207	7.98E-45	1.46E-112
Sharpea	1.24E-265	4.72E-250	1.71E-04
Slackia	1.87E-26	1.86E-10	7.14E-61
Solobacterium	1.42E-47	1.18E-17	1.55E-13
Sporobacter	9.77E-69	4.38E-65	6.75E-01
Sporobacterium	8.02E-19	8.57E-231	2.52E-193
Staphylococcus	8.63E-43	2.43E-20	5.31E-07
Streptococcus	1.52E-232	1.13E-238	1.58E-02
Subdoligranulum	1.42E-01	2.93E-46	2.30E-209
Succinivibrio	2.43E-298	3.41E-185	5.10E-177
Sutterella	3.13E-49	3.19E-101	3.75E-32
Synergistes	5.08E-230	1.01E-217	3.73E-03
Treponema	2.26E-11	3.25E-95	1.89E-136
Trueperella	3.43E-163	9.88E-152	3.70E-02
Turicibacter	0.00E+00	0.00E+00	0.00E+00
Unclassified_erysipelotrichaceae	4.28E-81	7.94E-211	1.18E-99
Unclassified_pasteurellaceae	0.00E+00	0.00E+00	6.36E-01
Unclassified_peptostreptococcaceae	0.00E+00	0.00E+00	5.63E-34
Veillonella	1.03E-165	1.38E-131	2.10E-09
Victivallis	3.37E-66	3.14E-45	2.59E-04
Weissella	2.23E-133	6.65E-20	4.92E-76

Wk15: week 15; vs: versus; NaN: no data.
