# Supplementary material for: Host contributes to longitudinal diversity of fecal microbiota in swine selected for lean growth
Source: Microbiome. 2018 Jan 4;6:4. doi: 10.1186/s40168-017-0384-1 (PMC5755158; doi:10.1186/s40168-017-0384-1)
Supplement: Supplementary file 6 — Sample flow between enterotypes through weaning, week 15, and off-test. (PDF 46 kb) [file 40168_2017_384_MOESM6_ESM.pdf]

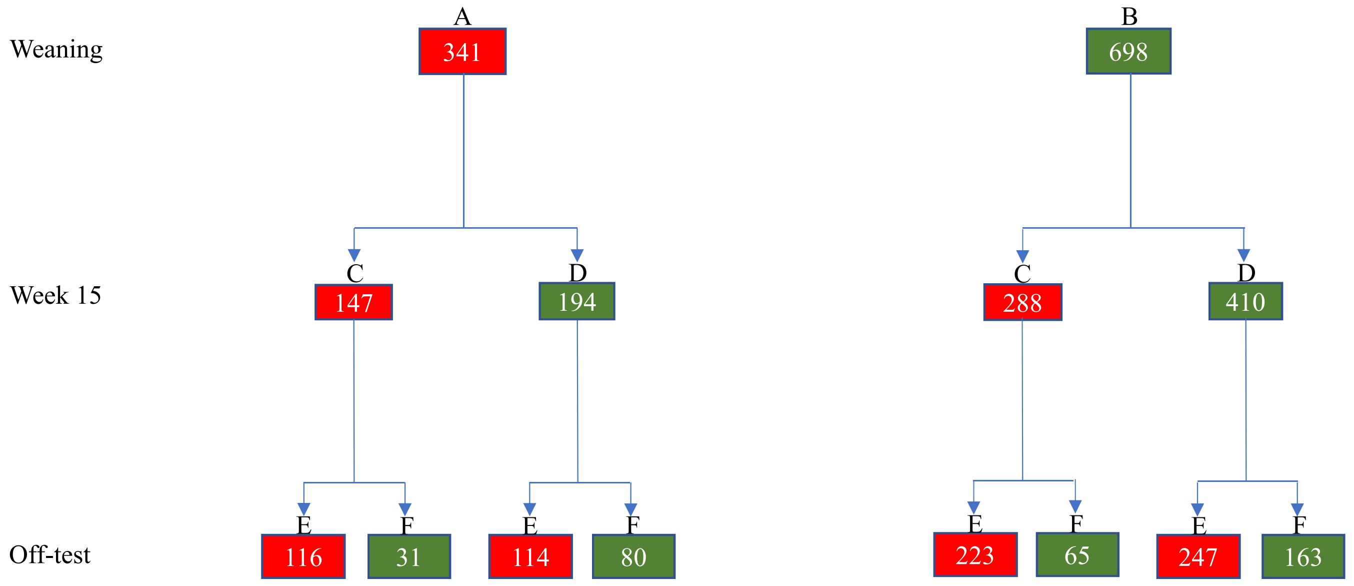

Figure S1. Sample flow between enterotypes through weaning, week 15, and off-test. A subset of 1039 animals that had samples collected at all 3 time points was used to create this graph. A and B are enterotypes at weaning; C and D are enterotypes at week 15; E and F are enterotypes at off-test.
