# Supplementary material for: Host contributes to longitudinal diversity of fecal microbiota in swine selected for lean growth
Source: Microbiome. 2018 Jan 4;6:4. doi: 10.1186/s40168-017-0384-1 (PMC5755158; doi:10.1186/s40168-017-0384-1)
Supplement: Supplementary file 7 — Distribution of samples in enterotypes by family. (PDF 43 kb) [file 40168_2017_384_MOESM7_ESM.pdf]

Table S13. Distribution of samples in enterotypes by family

Family	Enterotype						Total
	A	B	C	D	E	F	
1	7	21	11	17	19	9	84
2	13	25	21	17	23	15	114
3	10	23	23	10	28	5	99
4	9	22	14	17	26	5	93
5	16	23	11	28	18	21	117
6	7	33	12	28	23	17	120
7	13	26	15	24	23	16	117
8	18	18	13	23	24	12	108
9	9	25	17	17	27	7	102
10	9	30	13	26	31	8	117
11	10	23	15	18	21	12	99
12	14	26	25	15	26	14	120
13	10	24	10	24	26	8	102
14	18	18	15	21	31	5	108
15	17	23	11	29	25	15	120
16	21	17	13	25	26	12	114
17	18	15	16	17	29	4	99
18	10	37	17	30	29	18	141
19	8	23	9	22	16	15	93
20	8	32	18	22	37	3	120
21	16	25	17	24	20	21	123
22	8	33	15	26	27	14	123
23	10	31	15	26	26	15	123
24	15	20	17	18	26	9	105
25	15	26	19	22	23	18	123
26	10	27	21	16	31	6	111
27	12	25	17	20	17	20	111
28	10	27	15	22	22	15	111
