# Supplementary material for: Host contributes to longitudinal diversity of fecal microbiota in swine selected for lean growth
Source: Microbiome. 2018 Jan 4;6:4. doi: 10.1186/s40168-017-0384-1 (PMC5755158; doi:10.1186/s40168-017-0384-1)
Supplement: Supplementary file 8 — Effects and standard errors of family and time on alpha diversity estimated from model (1). (PDF 19 kb) [file 40168_2017_384_MOESM8_ESM.pdf]

Table S14. Effects and Standard Errors of family and time on alpha diversity estimated from Model 1

Factor		Effect	Standard Error
Sex	Female	0	0
	Male	-3.40E-02	1.26E-02
Birth site	1	0	0
	2	-5.05E-02	1.58E-02
	3	-1.64E-02	1.58E-02
Family	21	0	0
	16	-1.50E-01	8.44E-02
	15	4.77E-03	8.11E-02
	24	3.54E-02	8.40E-02
	22	-1.12E-02	8.44E-02
	17	-8.69E-02	8.39E-02
	27	8.45E-02	8.39E-02
	9	1.04E-01	8.54E-02
	5	-1.47E-01	9.16E-02
	28	-1.88E-01	8.54E-02
	10	-3.98E-02	8.60E-02
	1	3.53E-02	8.44E-02
	11	-9.57E-02	8.44E-02
	25	-6.02E-02	8.35E-02
	2	-9.15E-02	8.60E-02
	8	-7.87E-02	8.44E-02
	19	-3.85E-02	8.55E-02
	13	-2.75E-01	8.60E-02
	26	-7.69E-02	8.35E-02
	3	-4.74E-02	8.65E-02
	6	9.02E-02	8.30E-02
	4	-4.61E-02	8.35E-02
	20	-6.46E-02	8.54E-02
	23	-2.25E-02	8.78E-02
	14	-1.25E-01	8.35E-02
	12	-1.20E-02	8.55E-02
	18	-1.53E-01	8.35E-02
	7	5.13E-02	8.85E-02
Time	Weaning	0	0
	Week 15	1.73E+00	8.84E-02
	Off-test	1.82E+00	8.84E-02

The effect of Male was contrasted to Female; The effects of Birth sites 2 and 3 were contrasted to Birth site 1; The effect of each Family was contrasted to Family 22; The effect of Week 15 and Off-test were contrasted to Weaning.
