# Supplementary material for: Host contributes to longitudinal diversity of fecal microbiota in swine selected for lean growth
Source: Microbiome. 2018 Jan 4;6:4. doi: 10.1186/s40168-017-0384-1 (PMC5755158; doi:10.1186/s40168-017-0384-1)
Supplement: Supplementary file 9 — Proportion of variation explained by the first two principal components at different taxonomic levels and contribution of top members to the first two principal components, using unrarefied microbiome data. (PDF 47 kb) [file 40168_2017_384_MOESM9_ESM.pdf]

Table S15. Proportion of variation explained by first two principal components at different taxonomic levels, and contribution of top members to first two principal components, using raw data (unrarefied).

Taxonomic level	PC1	PC2	Contribution to PC1	Contribution to PC2
Phylum	93.69	3.58		
Firmicutes			75.04	3.77
Proteobacteria			8.01	54.20
Bacteroidetes			6.21	26.76

Taxonomic level	PC1	PC2	Contribution to PC1	Contribution to PC2
Class	62.69	12.42		
Clostridia			45.81	3.47
Gammaproteobacteria			12.90	4.54
Fusobacteriia			7.81	11.13
Bacteroidia			7.59	22.48
Erysipelotrichia			4.83	3.36
Epsilonproteobacteria			4.50	1.21

Taxonomic level	PC1	PC2	Contribution to PC1	Contribution to PC2
Order	58.12	11.61		
Clostridiales			40.12	7.07
Enterobacteriales			10.74	10.91
Fusobacteriales			7.13	8.97
Bacteroidales			6.76	7.85
Pasteurellales			5.98	6.85
Erysipelotrichales			4.27	1.68
Campylobacterales			4.12	3.83
Fibrobacterales			2.52	2.89

Taxonomic level	PC1	PC2	Contribution to PC1	Contribution to PC2
Family	31.99	12.79		
Enterobacteriaceae			12.46	7.52
Bacteroidaceae			6.58	0.91
Fusobacteriaceae			6.19	4.74
Enterococcaceae			5.62	2.52
Pasteurellaceae			5.38	2.68
Peptostreptococcaceae			5.16	3.81
Clostridiaceae			4.56	7.97
Streptococcaceae			4.30	4.29
Campylobacteraceae			3.11	1.92
Prevotellaceae			3.07	9.31

Taxonomic level	PC1	PC2	Contribution to PC1	Contribution to PC2
Genus	31.10	10.42		
Escherichia			6.26	4.94
Bacteroides			3.78	0.40
Fusobacterium			3.64	1.16
Peptostreptococcaceae			3.31	1.87
Enterococcus			2.95	1.94
Turicibacter			2.69	2.93
Clostridium			2.75	4.18
Streptococcus			2.64	1.35
Actinobacillus			2.57	0.47
Butyricimonas			2.50	1.42

Taxonomic level	PC1	PC2	Contribution to PC1	Contribution to PC2
Species	32.39	8.14		
Escherichia coli			2.22	1.09
Bacteroides fragilis			1.49	0.61
Bacteroides vulgatus			1.36	0.01
Campylobacter jejuni			1.27	0.26
Clostridium scindens			1.17	0.21
Clostridium bolteae			1.12	0.26

PC1 and PC2: principal component 1 and 2, respectively.
