# Supplementary material for: Host contributes to longitudinal diversity of fecal microbiota in swine selected for lean growth
Source: Microbiome. 2018 Jan 4;6:4. doi: 10.1186/s40168-017-0384-1 (PMC5755158; doi:10.1186/s40168-017-0384-1)
Supplement: Supplementary file 10 — Scatter plots of samples by principal component 1 (PC1) and principal component 2 (PC2) at six taxonomic levels, using unrarefied microbiome data. (PDF 447 kb) [file 40168_2017_384_MOESM10_ESM.pdf]

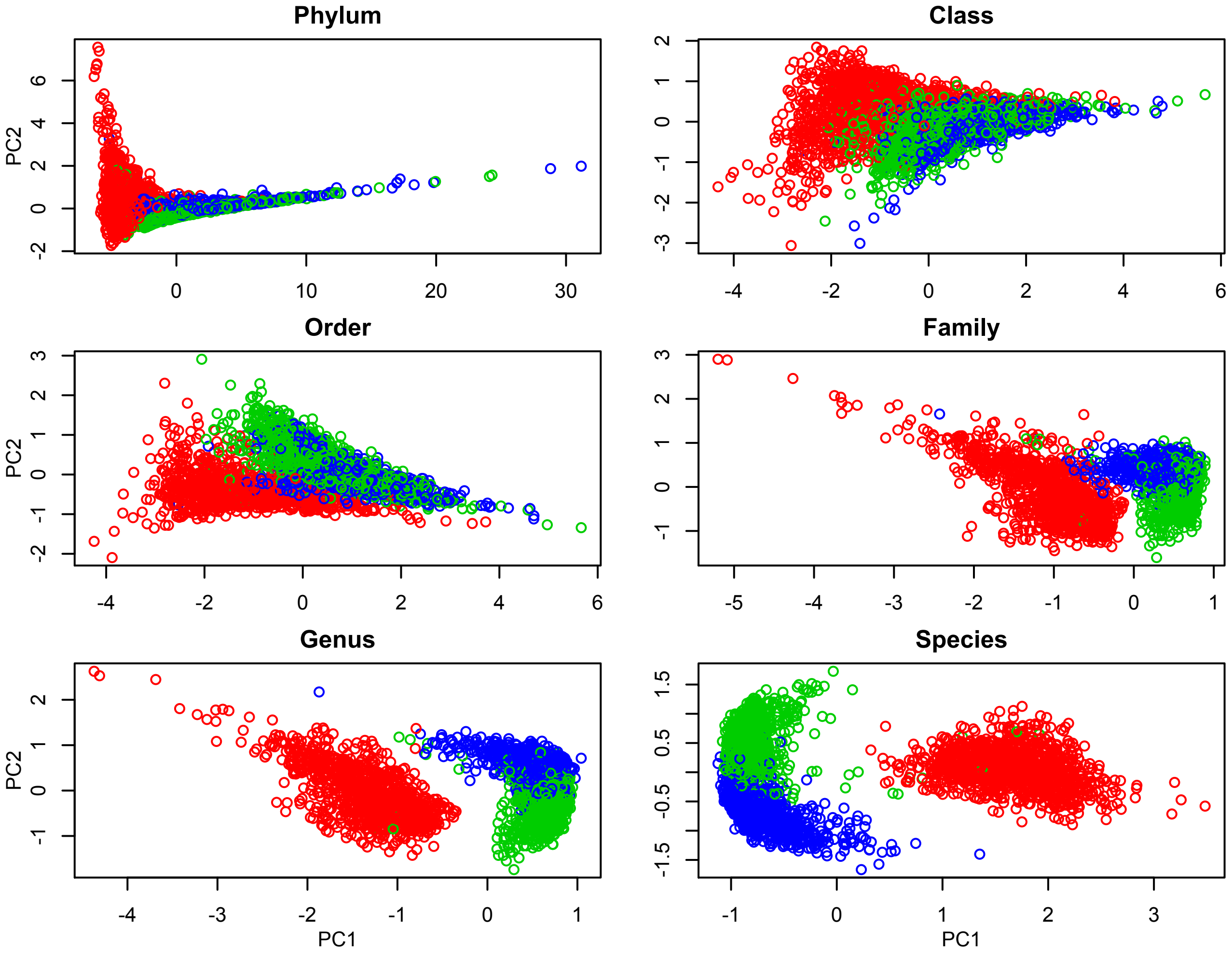

Figure S2. Scatter plots of samples collected at weaning (red circles), week 15 (green circles), and off-test (blue circles) by principal component 1 (PC1) and principal component 2 (PC2) at 6 taxonomic levels, using unrarefied microbiome data.
