# Supplementary material for: Host contributes to longitudinal diversity of fecal microbiota in swine selected for lean growth
Source: Microbiome. 2018 Jan 4;6:4. doi: 10.1186/s40168-017-0384-1 (PMC5755158; doi:10.1186/s40168-017-0384-1)
Supplement: Supplementary file 11 — Calinski-Harabasz indexes (CH) for number of potential clusters of samples at weaning, week 15, and off-test, using unrarefied microbiome data. (PDF 174 kb) [file 40168_2017_384_MOESM11_ESM.pdf]

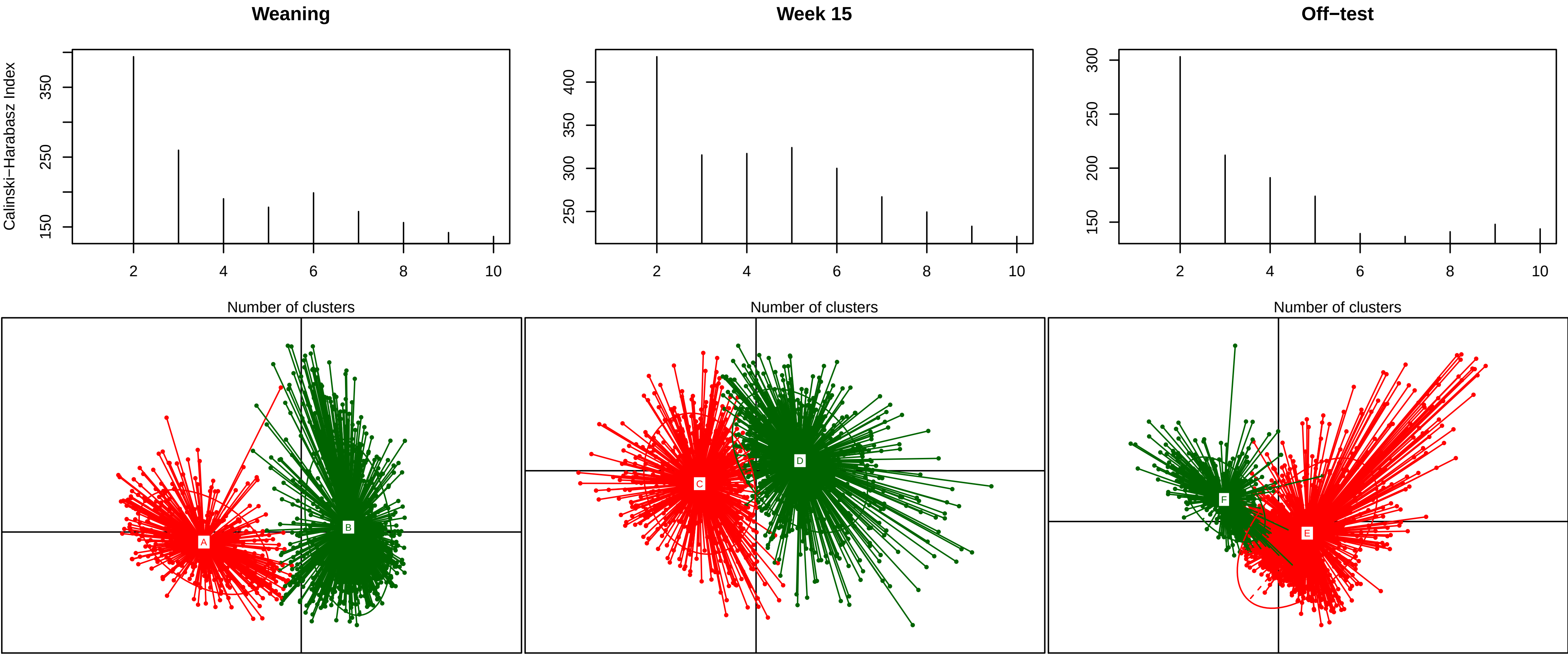

Figure S3. Calinski-Harabasz indexes (CH) for number of potential clusters of samples at weaning, week 15, and off-test, using unrarefied microbiome data. Highest CH value at each time point indicates optimal number of clusters/enterotypes. Samples at weaning formed 2 clusters, A and B. Samples at week 15 formed 2 clusters, C and D. Samples at off-test formed 2 clusters, E and F.
